# Antimicrobial and Antioxidant Secondary Metabolites from *Trifolium baccarinii* Chiov. (Fabaceae) and Their Mechanisms of Antibacterial Action

**DOI:** 10.1155/2021/3099428

**Published:** 2021-10-22

**Authors:** Donald Léonel Feugap Tsamo, Jean-De-Dieu Tamokou, Irene Chinda Kengne, Claudia Darille Jouogo Ngnokam, Mahamat Djamalladine Djamalladine, Laurence Voutquenne-Nazabadioko, David Ngnokam

**Affiliations:** ^1^Research Unit of Applied and Environmental Chemistry, Department of Chemistry, Faculty of Science, University of Dschang, P.O. Box 67 Dschang, Cameroon; ^2^Research Unit of Microbiology and Antimicrobial Substances, Department of Biochemistry, Faculty of Science, University of Dschang, P.O. Box 67 Dschang, Cameroon; ^3^Groupe Isolement et Structure, Institut de Chimie Moléculaire de Reims (ICMR), CNRS UMR 7312, Bat. 18 B.P. 1039, 51687 Reims Cedex 2, France

## Abstract

The treatment of infectious diseases with antimicrobial agents continues to present problems in modern-day medicine with many studies showing significant increase in the incidence of bacterial resistance to several antibiotics. The screening of antimicrobial activity of plant extracts and natural products has shown that medicinal plants are made up of a potential source of new anti-infective agents. The aim of this study was to evaluate the antimicrobial and antioxidant activities of extracts and compounds from the whole plant *Trifolium baccarinii* Chiov. and to determine their modes of antibacterial action. The plant extracts were prepared by maceration in organic solvents. The antimicrobial activities were evaluated using the broth microdilution method. The antioxidant activity was evaluated using the 2,2′-diphenyl-1-picrylhydrazyl radical (DPPH) and 2,2′-azino-bis(3-ethylbenzothiazoline-6-sulphonic acid) diammonium salt (ABTS) assays. The mechanisms of antibacterial action were determined by lysis, salt tolerance assays, and antioxidant enzyme activities. The cytotoxic effect on the erythrocytes was determined by a spectrophotometric method. Biochanin A, formononetin, luteolin, luteolin-4′-O-*β*-D-glucopyranoside, 4,7,2′-trihydroxy-4′-methoxyisoflavanol, sissotrin, 1-methyl-*β*-D-glucopyranoside, ononin, D-mannitol, and 3-O-*β*-D-glucuronopyranosylsoyasapogenol B were isolated from *Trifolium baccarinii*. The MeOH, EtOAc, and *n*-BuOH extracts as well as biochanin A, formononetin, luteolin, luteolin-4′-O-*β*-D-glucopyranoside, 4,7,2′-trihydroxy-4′-methoxyisoflavanol, and sissotrin from *Trifolium baccarinii* displayed the highest antimicrobial and antioxidant activities. The MeOH extract and 4,7,2′-trihydroxy-4′-methoxyisoflavanol exhibited antibacterial activity through the bacteriolytic effect and reduction of the antioxidant defenses in the bacterial cells. The present study portrays *Trifolium baccarinii* as a potential natural source of antibacterial, antifungal, and antioxidant agents.

## 1. Introduction

The treatment of infectious diseases with antimicrobial agents continues to present problems in modern-day medicine with many studies showing significant increase in the incidence of bacterial resistance to several antibiotics [1]. The expansion of antimicrobial resistance is accelerated by the selective pressure exerted by widespread use and misuse of antimicrobial drugs in both humans and food-producing animals. Antimicrobial resistance is a complex global public health challenge that leads to prolonged illness and increased mortality, increases the costs for the healthcare sector, and has an impact on animal health, which probably has an effect on food production [2]. As a remarkable case, over time, the original Gram-positive bacterium *Staphylococcus aureus* developed resistance towards a series of first-line, second-line, and even third-line antibiotics [3] to evolve into methicillin-resistant *S. aureus* (MRSA). Since MRSA is capable of resisting a beta-lactam group of antibiotics, this explains the emergent challenge in the treatment of this highly prevalent pathogen. The organism produces free radicals such as reactive oxygen species (ROS) which are formed during cell respiration and have a vital role in cell signaling [4]. It is generally known that the production of ROS increases during infections, enhancing pathogen clearance, as well as contributing to signaling cascades related to inflammation, cell proliferation, and immune responses [5]. However, high amounts of the free radicals produced during infection can cause oxidative stress, which further complicates the patient's prognosis.

Due to increased resistance of many microorganisms towards established antimicrobials, several scientific studies have been carried out on extracts and active principles isolated from medicinal plants [6] to be developed as future phytopharmaceuticals for the treatment of infectious diseases. The screening of antimicrobial activity of plant extracts and natural products has shown that medicinal plants are made up of a potential source of new anti-infective agents. Crude extracts from medicinal plants have demonstrated to be clinically effective and less toxic than the existing antibiotics [7]. Phytochemical compounds, particularly flavonoids and other natural compounds, play an important role in the defense against free radicals and pathogenic microorganisms [8, 9]. Hence, there is a justifiable need to explore for new and more potent antimicrobial/antioxidant compounds of natural origin to combat infectious diseases associated with drug-resistant microorganisms and oxidative stress.

Plants of the genus *Trifolium* are widespread throughout the world and represented in a total of four floristic regions: Neotropic, Paleotropic, Holarctic, and Capensis. They are generally small herbaceous plants, some creeping, which can be perennial, annual, or biennial. The species of the genus *Trifolium* are characterized by their capacity to fix atmospheric nitrogen through symbiotic bacteria hosted in their roots. The leaves are generally with three leaflets (sometimes four) and are at the origin of the name of the genus. The leaflets are almost always toothed, sometimes smudged at their center. *Trifolium baccarinii* Chiov. (Fabaceae) is an annual herb, glabrous or sparingly hairy in upper parts, 7-50 cm tall. The stems are upright or vertical, grooved and branched rooted at the nodes in an overgrazed area. It is a plant of montane grassland at elevations 1,600 m and over in West Cameroon and widespread in East Cameroon to Ethiopia and East Africa southwards to Democratic Republic of Congo and Tanzania. In the Cameroonian folk medicine, *Trifolium baccarinii* is used for the treatment of various diseases such as dermatosis, pulmonary infections, coughs, fevers, and rheumatisms. Some *Trifolium* species are reported to contain isoflavones, flavonoids, pterocarpans, saponins, coumarins, and tyramine [10–13]. The antioxidant, anti-inflammatory, and antimicrobial properties of some species of *Trifolium* have been determined [12–15]. To date, however, no scientific report is found in the literature regarding the antioxidant and antimicrobial activities of *Trifolium baccarinii*, although there is an ample ethnobotanical claim for these properties. As a continuing research directed at the biological properties of plants grown in Cameroon, this paper is designed to assess the antimicrobial and antioxidant activities of extracts and compounds from *Trifolium baccarinii* and establish their mechanisms of antibacterial action.

## 2. Materials and Methods

### 2.1. General Experimental Procedures

#### 2.1.1. NMR Analysis

The ^1^H and ^13^C-NMR spectra were recorded on a Bruker Avance III 600 spectrometer equipped with a cryoplatform (^1^H at 500 or 600 MHz and ^13^C at 125 or 150 MHz). 2D NMR experiments were performed using standard Bruker microprograms (Xwin-NMR version 2.1 software). All chemical shifts (*δ*) are reported in parts per million (ppm) with the solvent signal as a reference relative to TMS (*δ* = 0) as an internal standard, while the coupling constants (*J*) are given in hertz (Hz). Deuterated solvents, methanol (CD_3_OD), dimethyl sulfoxide (DMSO-*d*_6_), and chloroform (CDCl_3_) were used as solvents for the NMR experiments.

#### 2.1.2. Chromatographic Methods

Column chromatography was run on Merck silica gel (VWR, France) 60 (70–230 mesh) and gel permeation on Sephadex LH-20 (VWR, France), while TLC was carried out on silica gel GF254 precoated plates, and the spots were visualized by an UV lamp multiband UV-254/365 nm (Model UVGL-58, Upland, CA 91786, USA) followed by spraying with 50% H_2_SO_4_ followed by heating at 100°C.

### 2.2. Plant Collection

The whole plant *Trifolium baccarinii* Chiov. was collected in January 2016 in Dschang, western region of Cameroon. The botanical identification was carried out at the National Herbarium of Cameroon after comparison with the samples deposited at the reference number 2976/HNC. For the collection of the plant, no specific permit was required for the described field studies. For all locations/activities, no specific authorization was required. All locations of plant collection were not privately owned or protected in any way, and field studies did not involve threatened or protected species. The investigations concerning the plant were carried out following the recommendations of the Convention on Biological Diversity, signed by 150 government leaders at the Rio Earth Summit in 1992. Finally, all methods using a plant material were carried out in accordance with relevant guidelines and regulations.

### 2.3. Extraction and Fractionation

The plant material was air-dried at room temperature and ground into fine powder. The dried powder of *Trifolium baccarinii* (4.5 kg) was subjected to extraction at room temperature with methanol (3 × 20 L, 72 h) to yield 281 g of crude methanol extract after evaporation of solvent under reduced pressure. A part of crude extract (271 g) underwent a differential solubilization with EtOAc/H_2_O (500 mL/300 mL) followed by *n*-BuOH/H_2_O (500 mL/300 mL). After evaporation of each solvent under reduced pressure, we obtained 70 g of EtOAc and 54 g of *n*-BuOH extracts.

### 2.4. Isolation of Compounds

A part of the EtOAc extract (65 g) of *Trifolium baccarinii* was subjected to silica gel column chromatography using *n*-hexane-EtOAc (100 : 0⟶0 : 100) followed by EtOAc-MeOH (90 : 10⟶80 : 20) gradient as an eluting system. Sixty-six fractions of 400 mL were collected and combined on the basis of their TLC profiles to give fourteen major fractions: A (1), B (2-6), C (7-10), D (11-14), E (15-20), F (21-25), G (26-31), H (32-34), I (35-40), J (41-46), K (47-52), L (53-58), M (59-60), and N (61-66). Purification of fraction G (8.0 g) on silica gel column chromatography with *n*-hexane-EtOAc (90 : 10) as an eluent yielded compounds **1** (27 mg) and **2** (35 mg). Silica gel column chromatography of fraction H (10.0 g) eluted with *n*-hexane-EtOAc (80 : 20) gave compound **3** (10 mg). Fraction K (5 g) was subjected to silica gel column chromatography eluted with EtOAc to acquire seven subfractions (K_1_-K_7_). Compound **4** (10 mg) was obtained from subfraction K_3_ (250 mg) after Sephadex LH-20 column chromatography using MeOH as an eluent. Fraction D (4.2 kg) was run through silica gel column chromatography and eluted with *n*-hexane-EtOAc (93 : 7) to yield four subfractions (D_1_-D_4_). Further purification of subfraction D_2_ on silica gel column chromatography eluted with *n*-hexane-EtOAc (95 : 5) yielded compound **5** (30 mg). The purification of fraction J (3.5 g) on Sephadex LH-20 column chromatography produced two subfractions J_1_ and J_2_. Subfraction J_2_ (600 mg) was further purified on silica gel column chromatography with *n*-hexane-EtOAc (20 : 80) to give compound **6** (20 mg).

As the EtOAc extract, a part of the *n*-BuOH extract (50 g) of *Trifolium baccarinii* was subjected to silica gel column chromatography using the mixture EtOAc-MeOH (100 : 0⟶40 : 60) as an elution system by increasing polarity. Seventy-six (76) fractions of 400 mL were collected and combined on the basis of their TLC profiles in four major fractions: A (1-7), B (8-23), C (24-41), and D (42-68). Purification of fraction B (5 g) on silica gel column chromatography with EtOAc as an eluent mainly led to compound **6** (580 mg), as a major constituent of this fraction, and to compounds **7** (20 mg) and **8** (16 mg). Fraction C (4 g) was purified over silica gel column chromatography eluted with EtOAc-MeOH-H_2_O (90 : 10 : 5) to give compound **9** (20 mg). Fraction D (3 g) was subjected to column chromatography over silica gel using the mixture EtOAc-MeOH-H_2_O (90 : 10 : 5) followed by the mixture EtOAc-MeOH-H_2_O (80 : 20 : 10) as eluents to give two subfractions D_1_ and D_2_. The subfraction D_2_ (1 g) was subsequently purified on silica gel column chromatography using EtOAc-MeOH-H_2_O (80 : 10 : 10) as the system to give also two other subfractions D_2-1_ and D_2-2_. Purification of subfraction D_2-2_ (200 mg) on Sephadex LH-20 column chromatography using MeOH as an eluent yielded compound **10** (20 mg).

### 2.5. Antimicrobial Assay

#### 2.5.1. Microorganisms

The antimicrobial activity was performed against five bacterial and two fungal species. The selected microorganisms were the Gram-positive (*Staphylococcus aureus* ATCC25923, methicillin-resistant *Staphylococcus aureus* 03 (MRSA03), and methicillin-resistant *Staphylococcus aureus* 04 (MRSA04)) and Gram-negative (*Pseudomonas aeruginosa* ATCC27853 and *Escherichia coli* S2(1)) bacteria and yeast strains of *Candida albicans* ATCC10231 and *Cryptococcus neoformans* H99. These microorganisms were taken from our laboratory collection. The fungal and bacterial strains were conserved on Sabouraud Dextrose Agar (SDA, Conda, Madrid, Spain) and nutrient agar (NA, Conda) slants, respectively.

#### 2.5.2. Determination of Minimum Inhibitory Concentration (MIC) and Minimum Microbicidal Concentration (MMC)

The antimicrobial activity was investigated by determining the minimum inhibitory concentrations (MICs), minimum bactericidal concentrations (MBC), and minimum fungicidal concentrations (MFCs). MICs of extracts/compounds were monitored using the broth microdilution method [16]. Dimethyl sulfoxide (DMSO, Fisher Chemicals) was used to dissolve the test sample to produce a stock solution that was twofold serially diluted in Mueller-Hinton broth (MHB) for bacteria and Sabouraud Dextrose Broth (SDB) for fungi to obtain a concentration range of 4096 to 0.25 *μ*g/mL. One hundred microliters of each concentration was introduced into a well (96-well microplate) containing 90 *μ*L of SDB or MHB and 10 *μ*L of inoculum (at 1 × 10^6^ CFU/mL for bacteria and 1 × 10^5^ spores/mL for yeasts) which were added to obtain a final concentration range of 2048 to 0.125 *μ*g/mL. Plates were covered and incubated on the shaker at 37°C, 30°C, and 30°C during 24 h, 48 h, and 72 h for bacteria, *Candida albicans*, and *Cryptococcus neoformans*, respectively. The MICs were visually assessed after the incubation period and were considered the lowest sample concentration that inhibits the growth or the visual growth of the organism.

For the minimum microbicidal concentration (MMC) determination, 10 *μ*L aliquots from each well that showed no growth of microorganism was plated on Mueller-Hinton agar or Sabouraud Dextrose Agar and incubated as described above. The lowest concentration that showed no growth after subculturing was considered the MBC or MFC. The positive controls were oxacillin (Sigma-Aldrich, Steinheim, Germany) for bacteria and nystatin (Sigma-Aldrich, Steinheim, Germany) for yeasts, and the negative control was broth with 20 *μ*L of DMSO. The assay was carried out in triplicate.

#### 2.5.3. Antibacterial Mechanism Studies

The mechanisms of antibacterial action were determined by lysis, salt tolerance assays, and antioxidant enzyme activities.

*(1) Bacteriolytic Assay*. The bacteriolytic activity of the methanol extract and compound **5**, which exhibited the highest antimicrobial activities, was performed against *Pseudomonas aeruginosa* ATCC27853 and *Staphylococcus aureus* ATCC25923 using the time-kill kinetic method as previously described [17] with some modifications. Full growth of bacterium in MHB was diluted 100 times and incubated at 37°C to produce an OD_600_ of 0.8 as a starting inoculum. Sample solutions were added to the starting bacterial suspension to give a final concentration of 2xMIC and incubated at 37°C under agitation at 150 rpm. After the incubation period corresponding to 0, 15, 30, 60, 120, and 240 min, 100 *μ*L was pipetted from each tube and the optical density was recorded at 600 nm using the Biobase UV-Vis spectrophotometer. Corresponding dilutions of test samples were used as blanks. Oxacillin was used as a positive control, and the tubes without an extract/compound served as negative controls. All the measurements were done in triplicate and repeated three times.

*(2) Loss of Salt Tolerance in Staphylococcus aureus*. The ability of *Staphylococcus aureus* ATCC25923 and MRSA03 cells treated with the methanol extract and compound **5** to grow on Mueller-Hinton agar (MHA) supplemented with NaCl was investigated. In preliminary experiments, untreated suspensions of *Staphylococcus aureus* were plated on MHA supplemented with NaCl from 40 to 100 g/L. Plates were incubated. After incubation, the resulting colonies were counted. Concentrations of NaCl, 50, 60, and 70 g/L, which modestly compromised the colony-forming abilities of untreated microorganisms, were selected. For further experimentation steps, suspensions of bacteria were prepared as previously described and treated with the MeOH extract or compound **5** at 1/2xMIC, MIC, and 2xMIC. After 1 h incubation, samples were removed, serially diluted, and inoculated onto MHA and MHA-NaCl (50, 60, and 70 g/L). Bacterial culture without a sample was used as a control for each MHA-NaCl plate. Upon incubation, the numbers of CFU per milliliter on each MHA-NaCl plate were compared to those on the MHA plate, and the result was expressed as a percentage [18].

*(3) Antioxidant Enzyme Activities*. For evaluation of catalase and superoxide dismutase (SOD) activities, *Staphylococcus aureus* ATCC25923 and MRSA03 (1.5 × 10^8^ CFU/mL, 500 *μ*L) cultures from the late exponential growth phase were treated with MIC and 1/2xMIC of the methanol extract (500 *μ*L), compound **5** (500 *μ*L), and oxacillin (500 *μ*L) solutions and incubated at 37°C for 24 h. The suspension was centrifuged at 3000 rpm for 5 min to separate the supernatant. The pellet was washed twice with PBS and resuspended in 500 *μ*L of cell lysate buffer (1 mM EDTA, 10 Mm Tris-HCl, 0.1% Triton-X-100, and 150 mM NaCl) [19] and incubated at 37°C for 1 h. Contents were then centrifuged at 3000 rpm for 5 min, and the supernatant was collected for enzyme activity assays.

*Catalase activity.* This was assessed by using a kit (Sigma, catalogue no. CAT100) with a cell lysate. Briefly, 750 *μ*L of assay buffer (50 mM) was mixed with 25 *μ*L of 50 mM H_2_O_2_ and 10 *μ*L of cell lysate. The mixture was incubated for 5 min. After this, reaction was terminated by the addition of 900 *μ*L of stop solution (15 mM sodium azide) and the content was thoroughly mixed. Then, 10 *μ*L of reaction mixture was taken into a separate tube and mixed with 1 mL of colour reagent (2 mM 3,5-dichloro-2-hydroxybenzenesulfonic acid and 0.25 mM 4-aminoantipyrine) and incubated for 15 min. The absorbance was monitored at 520 nm, and the catalase activity was calculated based on the following equation: [Δ*μ* moles (H2O2) × *d* × 100)/*V* × *t*], where Δ*μ* moles (H_2_O_2_) is the difference in amount of H_2_O_2_ added to the reaction mixture between blank and given samples, *d* is the dilution of the original sample for catalase reaction, *V* is the sample volume in catalase reaction, and *t* is the reaction duration (min).

*Superoxide dismutase (SOD) activity.* This was determined using a kit (Sigma, catalogue no. 19160) in the cell lysate. The cell lysate (20 *μ*L) was mixed with working solution of water soluble tetrazolium salt (WST, 200 *μ*L) and enzyme solution (20 *μ*L). The reaction mixture was incubated in the dark at 37°C for 20 min, and the absorbance was read at 450 nm on a BioTek Synergy 2 multiplate reader. The SOD activity was calculated based on the following formula: [(*A*_Blank1_–*A*_Blank3_) − (*A*_Sample_–*A*_Blank2_)/(*A*_Blank1_–*A*_Blank3_) × 100], where Blank 1 contains ultrapure water, WST solution, and enzyme solution, Blank 2 contains sample solution, WST solution, and dilution buffer, and Blank 3 contains ultrapure water, WST solution, and dilution buffer.

### 2.6. Antioxidant Assay

#### 2.6.1. Gallic Acid Equivalent Antioxidant Capacity (GEAC) Assay

The GEAC test was performed as described previously [20] with some slight modifications. The laccase was purified from *Sclerotinia sclerotiorum* according to the described protocol [21]. 20 *μ*L of laccase (1 mM stock solution), 20 *μ*L of test sample, 10 *μ*L of ABTS (2,2′-azino-bis(3-ethylbenzothiazoline-6-sulfonic acid) (74 mM stock solution), and 950 *μ*L of acetate buffer (pH = 5.0, 100 mM) were added in a quartz cuvette. The concentrations of the samples in the assay mixture were 800, 400, 200, 100, and 10 *μ*g/mL for the extracts and 200, 100, 50, 25, and 12.5 *μ*g/mL for the isolated compounds. The content of the generated ABTS^●+^ radical was measured at 420 nm after 240 s reaction time and was converted to gallic acid equivalents by the use of a calibration curve (Pearson's correlation coefficient: *r* = 0.998) constructed with 0, 4, 10, 14, 28, 56, and 84 *μ*M gallic acid standards rather than Trolox. Experiments were done in triplicate and repeated three times with similar results.

#### 2.6.2. Diphenyl-1-picrylhydrazyl (DPPH) Free Radical Scavenging Assay

The free radical scavenging activity of extracts and compounds was evaluated according to the described method [22]. The EC_50_ (*μ*g/mL), which is the amount of sample necessary to inhibit by 50% the absorbance of free radical DPPH, was calculated [22]. Vitamin C was used as a standard control. All the analyses were carried out in triplicate and repeated three times.

### 2.7. Haemolysis Assay

Wistar rats (*Rattus norvegicus*) aged 10–12 weeks and weighing 220 to 250 g were randomly selected from our colony. Efforts were also made to minimize animal suffering and to reduce the number of animals used in the experiment. Intraperitoneal injection of the mixture of ketamine (50 mg/kg body weight, BW) and xylazine (10 mg/kg BW) was used to anaesthetize the rats in a dose that is commonly used for operation purposes. And subsequently, the unconscious animals were quickly operated on and the whole blood (10 mL) was collected by cardiac puncture into a conical tube containing Ethylenediaminetetraacetic Acid (EDTA) as an anticoagulant. Red blood cells were harvested by centrifugation at room temperature for 10 min at 1,000 × *g* and were washed three times in PBS buffer^23^. The haemolysis assay was evaluated as previously described [23].

### 2.8. Statistical Analysis

Data were analyzed by one-way analysis of variance followed by the Waller-Duncan post hoc test. The experimental results were expressed as the mean ± standard deviation (SD). Differences between groups were considered significant when *p* < 0.05. All analyses were performed using the Statistical Package for the Social Sciences (SPSS, version 12.0) software.

## 3. Results

### 3.1. Chemical Analysis

**Figure 1:**
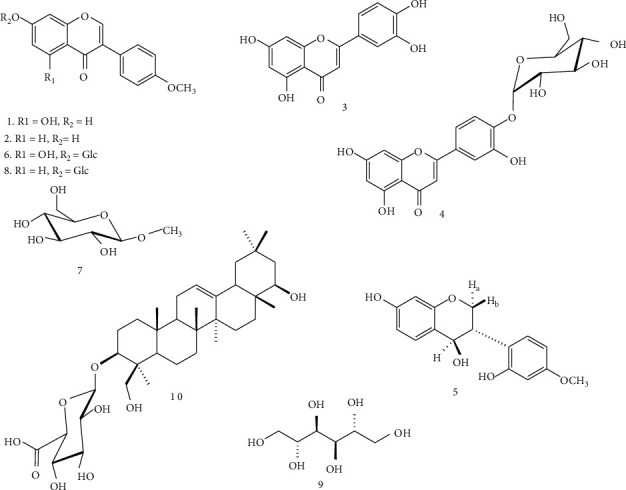
Chemical structures of compounds isolated from *Trifolium baccarinii* (**1**–**10**). **1**: biochanin A; **2**: formononetin; **3**: luteolin; **4**: luteolin-4′-O-*β*-D-glucopyranoside; **5**: 4,7,2′-trihydroxy-4′-methoxyisoflavanol; **6**: sissotrin; **7**: 1-methyl-*β*-D-glucopyranoside; **8**: ononin; **9**: D-mannitol; and **10**: 3-O-*β*-D-glucuronopyranosylsoyasapogenol B.

The purification of the EtOAc and *n*-BuOH extracts from *Trifolium baccarinii* led to the isolation of ten known compounds (Figure 1). The structures of these compounds were established on the basis of spectroscopic data: ^1^H and ^13^C NMR, ^1^H-^1^H COSY, HSQC, HMBC, and ROESY (supplementary materials/figures (available here)). The direct comparison with published information led us to identify these compounds as follows: biochanin A (**1**) [24]; formononetin (**2**) [24]; luteolin (**3**) [25]; luteolin-4′-*O*-*β*-D-glucopyranoside (**4**) [26]; 4,7,2′-trihydroxy-4′-methoxyisoflavanol (**5**) [27]; sissotrin (**6**) [28]; 1-methyl-*β*-D-glucopyranoside (**7**) [29]; ononin (**8**) [30]; D-mannitol (**9**) [31]; and 3-*O*-*β*-D-glucuronopyranosylsoyasapogenol B (**10**) [32].

### 3.2. Antimicrobial Activity

The antimicrobial properties of extracts and isolated compounds from *Trifolium baccarinii* were evaluated against both pathogenic bacteria and fungi by determining their minimum inhibitory concentration (MIC) values, and the results are depicted in Table 1. The MeOH, EtOAc, and *n*-BuOH extracts from *Trifolium baccarinii* were effective in inhibiting the growth of all tested yeasts and Gram-positive and Gram-negative bacteria with MIC values in the range of 32–512 *μ*g/mL. The MIC values of MeOH and EtOAc extracts were in the range of 32-64 *μ*g/mL, whereas the *n*-BuOH extract was active in the range of 128-512 *μ*g/mL towards the tested bacteria and yeasts. This result suggests that the *n*-BuOH extract was lesser active than the MeOH and EtOAc extracts. Indeed, a lowest MIC value indicates a largest antimicrobial agent as fewer samples are required to inhibit the growth of the microorganism. The lowest MIC value of 8 *μ*g/mL was recorded on *Cryptococcus neoformans* with compound **3** and on *Pseudomonas aeruginosa*, *Escherichia coli*, *Staphylococcus aureus* ATCC25923, *Candida albicans*, and *Cryptococcus neoformans* with compound **5** whereas the lowest MMC value was obtained on *Pseudomonas aeruginosa*, *Staphylococcus aureus* ATCC25923, and *Cryptococcus neoformans* with compound **5**. However, the highest MIC value of 512 *μ*g/mL was recorded with the *n*-BuOH extract against MRSA04, while the highest MMC value of 2048 *μ*g/mL was obtained on MRSA03 with the *n*-BuOH extract.

Compounds **1**–**6** and **8** were active against all the yeasts and Gram-positive and Gram-negative bacteria. By contrast, compound **7** was not active against the tested microorganisms whereas compounds **9** and **10** exhibited low activity and only showed weak inhibition against *Pseudomonas aeruginosa*, *Escherichia coli*, *Staphylococcus aureus* ATCC25923, and *Cryptococcus neoformans*. Compound **5** (MIC = 8–32 *μ*g/mL) was the most active compound followed in decreasing order by compound **3** (MIC = 8–32 *μ*g/mL), compound **4** (MIC = 16– 64 *μ*g/mL), compound **1** (MIC = 32–64 *μ*g/mL), compound **2** (MIC = 64–128 *μ*g/mL), compound **6** (MIC = 32–256 *μ*g/mL), compound **8** (MIC = 64–256 *μ*g/mL), compound **9** (MIC ≥ 128 *μ*g/mL), compound **10** (MIC ≥ 256 *μ*g/mL), and compound **7** (MIC > 256 *μ*g/mL). As shown in Table 1, oxacillin and nystatin used as standard drugs were more potent than compound **5** against yeasts and Gram-positive and Gram-negative bacteria with the exception of *E. coli* where oxacillin was less active compared with compound **5**. The most susceptible strains towards the tested samples were *Pseudomonas aeruginosa*, *Escherichia coli*, *Staphylococcus aureus* ATCC25923, and *Cryptococcus neoformans* whereas the most resistant strains were MRSA03, MRSA04, and *Candida albicans*.

### 3.3. Mechanism of Antibacterial Activity

#### 3.3.1. Bacteriolytic Activity

**Figure 2:**
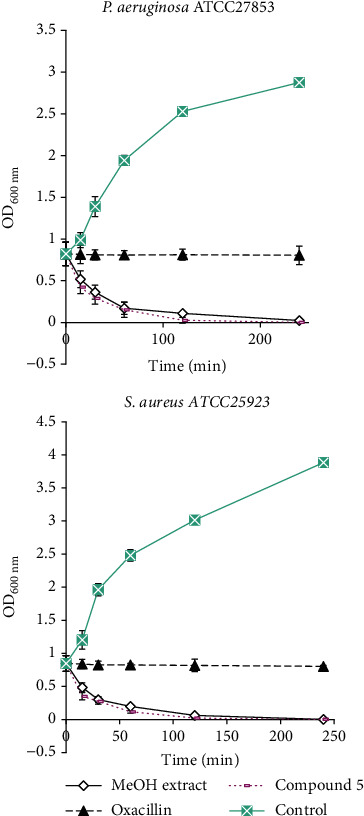
Bacteriolytic effect of the MeOH extract and compound **5** against *Pseudomonas aeruginosa* and *Staphylococcus aureus*. Results represent the mean ± standard deviation of the triplicate OD at each incubation time.

The result on the bacteriolytic activity showed a decrease in the optical density of bacterial suspensions treated with the MeOH extract and compound **5** as a function of time (Figure 2). Most of the decrease in the optical density was observed during the first periods of incubation (30, 60, and 120 min) followed by a slight decrease in the optical density after 120 min of incubation. After 240 min, the MeOH extract and compound **5** induced a decline in cell turbidity of 97.08 and 99.85% in *Pseudomonas aeruginosa* suspension and of 99.79 and 99.87% in *Staphylococcus aureus* ATCC25923 suspension, respectively, compared to the 0 time value, indicating the lysis of bacterial cells. Treatment with oxacillin had no effect (decline in cell turbidity of 2% to the 0 time value).

#### 3.3.2. Loss of Salt Tolerance in *Staphylococcus aureus* ATCC25923

**Figure 3:**
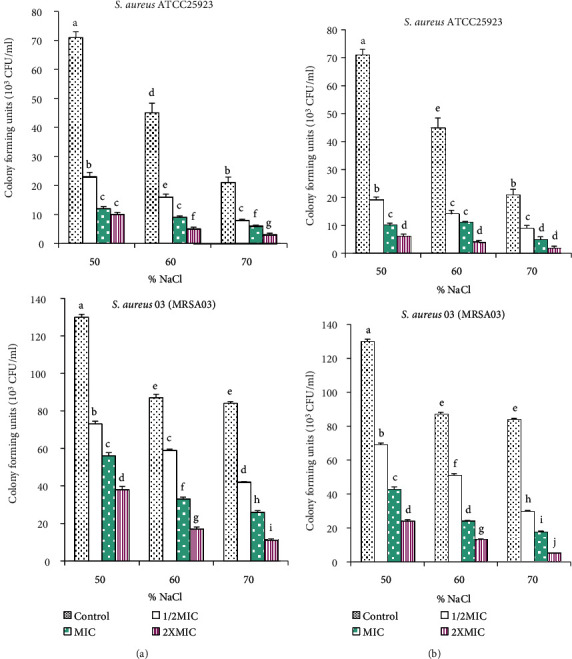
Effect of the *Trifolium baccarinii* methanol extract (a) and compound **5** (b) on the reduction of salt tolerance of *Staphylococcus aureus*. MIC: minimum inhibitory concentration; bars represent the mean ± standard deviation of the triplicate CFU. For the same figure, values with different letters are significantly different at *p* < 0.05 according to the Waller-Duncan test.

The effect of different concentrations of the *Trifolium baccarinii* MeOH extract (A) and compound **5** (B) on the reduction of salt tolerance of *Staphylococcus aureus* is shown in Figure 3. This figure shows that when the bacteria pretreated with samples were inoculated on culture media supplemented with different concentrations of NaCl, a significant decrease in the number of colony-forming units was observed depending on the pretreatment concentrations of the MeOH extract/compound **5**. Compared to other concentrations, the largest reductions in the number of colonies formed were observed on culture medium supplemented at 70% NaCl with two times the MICs.

#### 3.3.3. Antioxidant Enzyme Activities

**Figure 4:**
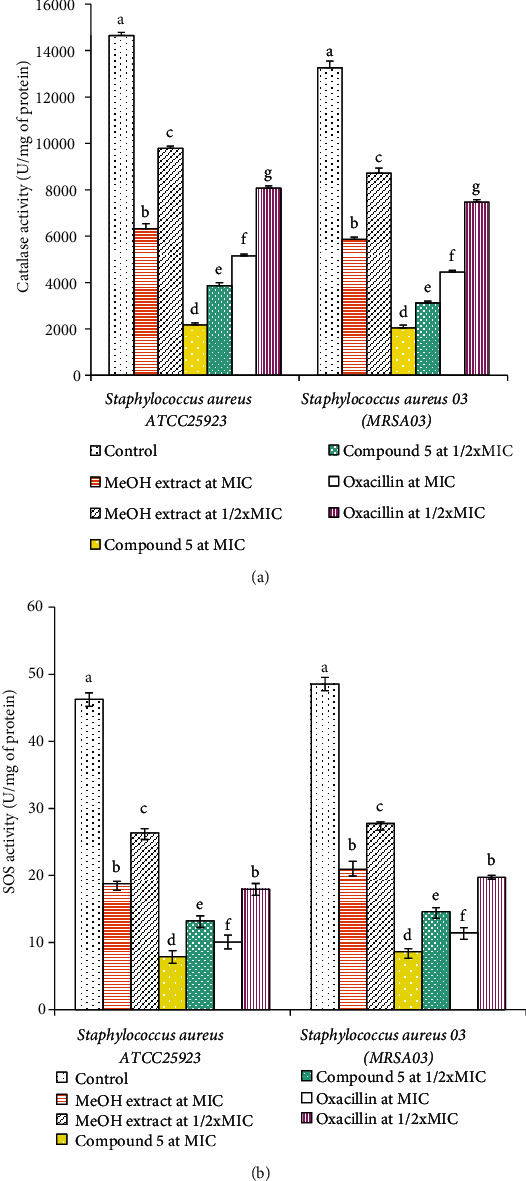
Antioxidant catalase (a) and superoxide dismutase (b) activities in *Staphylococcus aureus* ATCC25923 and methicillin-resistant *Staphylococcus aureus* 03 (MRSA03) treated with the MeOH extract and compound **5**. Bars represent the mean ± SD of three independent experiments carried out in triplicate. For the same microorganism and enzyme, values earmarked by different superscript letters (a–g) are significantly different according to one-way ANOVA and the Waller-Duncan test (*p* < 0.05).

Cell lysates treated with the methanol extract, compound **5**, and oxacillin showed significant concentration-dependent decreases in catalase and SOD activities compared to untreated cell lysates (negative control) (Figure 4). Also, treatment with MIC of the MeOH extract, compound **5**, and oxacillin displayed the most significant decreases in catalase and SOS activities when compared to their 1/2xMIC treatment against *Staphylococcus aureus* ATCC25923 and MRSA03. The effect of compound **5** on the catalase and SOS activities was significantly greater than those of oxacillin and MeOH extract.

### 3.4. Antioxidant Activity

The extracts and their isolated compounds were evaluated for their antioxidant activity using DPPH and TEAC methods (Table 2). The DPPH^•^ and ABTS^•+^ radical scavenging activities were observed in all the extracts. The lowest IC_50_ value reflects the highest DPPH radical scavenging activity whereas the largest gallic acid equivalent antioxidant capacity represents the highest ABTS^•+^ radical scavenging activity. According to the results obtained, the *n*-BuOH extract was the most potent antioxidant extract followed in decreasing order by the MeOH extract and EtOAc extract. Compounds **7**, **9**, and **10** were found to be inactive in both DPPH and TEAC assays. Compound **4** was the most active antioxidant compound followed in decreasing order by compounds **1**, **2**, **6**, **3**, **5**, and **8**.

### 3.5. Haemolytic Activity

The haemolytic activity of extracts and isolated compounds from *Trifolium baccarinii* against red blood cells (RBCs) was investigated using Triton X-100 as a positive control. The positive control demonstrated about 100% lysis as compared to the phosphate buffer saline (PBS) that showed no lysis of RBCs. Interestingly, none of the tested extracts and compounds showed cytotoxic activity against RBCs at concentrations up to 2048 *μ*g/mL for the extracts and 256 *μ*g/mL for the isolated compounds (results not shown).

## 4. Discussion

The antimicrobial activity of the MeOH extract was comparable with that of the EtOAc extract but higher than that of the *n*-BuOH extract, indicating that fractionation decreased the antimicrobial activity of the *n*-BuOH extract and did not affect that of the EtOAc extract. The findings of the present study revealed that the MeOH, *n*-BuOH, and EtOAc extracts from *Trifolium baccarinii* showed different degrees of antimicrobial activities against bacterial and fungal strains. Differences observed in the antimicrobial activities of extracts can be linked to the differences in their chemical composition whereas variations in the susceptibility of tested microorganisms can be explained by the genetic differences between the strains. Our present results reveal the potential of *Trifolium baccarinii* as a source of antibacterial and antifungal drugs and provide scientific evidence for its use in folk medicine for the treatment of various infectious diseases. To the best of our knowledge, no previous publications have reported the antibacterial and antifungal activities of *Trifolium baccarinii*. So, this plant can be used as a novel therapeutic agent to prevent the progress of various infectious diseases particularly those caused by the tested microorganisms. In addition, this is the first study to use spectroscopic methods for the identification of chemical constituents from *Trifolium baccarinii* in which compounds have been identified for the first time in this plant. According to the antimicrobial cutoff points defined in the literature for the plant extract [33], the MeOH and EtOAc extracts of *Trifolium baccarinii* were highly active (MIC < 100 *μ*g/mL) against all the tested microorganisms whereas the *n*-BuOH extract was significantly active (100 ≤ MIC ≤ 512 *μ*g/mL) against the tested microorganisms.

The microbicidal properties of extracts and isolated compounds against susceptible strains were analyzed by the minimum microbicidal concentration (MMC) assay. An antimicrobial agent is considered microbicidal if the MMC is not more than fourfold higher than the MIC, i.e., MMC/MIC ≤ 4 [22]. The MeOH, EtOAc, and *n*-BuOH extracts as well as compounds **1–6** and **8** were microbicidal (MMC/MIC ≤ 2) against the susceptible microorganisms with exception of the *n*-BuOH extract against *Escherichia coli* and MRSA04 and compounds **6** and **8** against MRSA03, MRSA04, *Candida albicans*, and *Cryptococcus neoformans*, with the MMC values being eightfold higher than the MIC indicating bacteriostatic character. These results suggest that the bacteriostatic/fungistatic and bactericidal/fungicidal activities of the *n*-BuOH extract and compounds **6** and **8** are dependent on the microbial strain. This behaviour is different from standard antibiotics, oxacillin and nystatin, which displayed microbicidal activities (MMC/MIC ≤ 4) against all the tested microorganisms.

Considering the antimicrobial cutoff points of pure compounds defined in a previous report [33], the antimicrobial activities of test compounds could be considered significant/moderate for compounds **3** and **5**; moderate for compounds **1** and **4**; moderate/low for compounds **2**, **6**, and **8**; low/not active for compounds **9** and **10**; and not active for compound **7** against specific microorganism.

All the compounds that were found to be active in the present study are members of saponin and flavonoid groups. Although saponin and flavonoid compounds have been reported to possess antibacterial and antifungal activities [9, 21], no study has reported the activity of compounds **4-6**, **8**, and **10** on the types of pathogenic microbial strains used in the present study. In comparison with the current results, previous studies have shown that biochanin A has an inhibitory effect against fungi [34] and a moderate activity against *S. aureus*, *Salmonella* spp., *Shigella* spp., *Klebsiella* spp., *Pseudomonas* spp., *Vibrio cholera*, *V. parahaemolyticus* [35], *Mycobacterium smegmatis* [36], *Clostridium clostridioforme*, and *Clostridium perfringens* [37] when tested at concentrations ranging from 25 to 1024 *μ*g/mL. Similar to our results, the isolated isoflavone formononetin inhibited the growth of yeasts (*C. albicans*, *C. tropicalis*, and *C. neoformans*) and Gram-positive (*S. aureus*, *S. epidermidis*) and Gram-negative (*P. aeruginosa*) bacteria with a minimum inhibitory concentration of 200 *μ*g/mL for the bacteria and 25 *μ*g/mL for the yeasts [38]. The MIC values of luteolin were remarkably weaker than those of luteolin against methicillin-resistant *Staphylococcus aureus* and methicillin-sensitive *S. aureus* strains (MIC = 62.5 *μ*g/mL) from a previous study [39]. The antibacterial mechanism of luteolin against *Staphylococcus aureus* is due to the inhibition of the activity of DNA topoisomerases I and II, which resulted in some decrease in the nucleic acid and protein synthesis [40]. The mechanism of action of saponin (**10**) is not fully understood, but it may involve membrane disruption by lipophilic compounds [41]. The mechanism of action of flavonoids (**1**–**6**, **8**-**9**) is still to be studied; nevertheless, their activity may be due to the disruption of microbial membranes and their ability to complex bacterial cell walls and extracellular and soluble proteins [42]. With regard to the structure-activity relationship analysis, it is suggested that the number and position of hydroxyl, sugar, and methoxy groups in the flavonic skeleton of compounds **1–4**, **6**, and **8** as well as on the triterpene skeleton **10** are responsible for different degrees of antibacterial, antifungal, and antioxidant activities observed. Compared with traditional antibiotics with a single target, the bioactive compounds have the advantage that they have multiple action targets on the tested bacteria and fungi, which is also the reason why natural products do not easily produce drug resistance.

Our results demonstrate that the *n*-BuOH extract is the most potent antioxidant extract among the extracts whereas compounds **1–4** and **6** are the most active antioxidant compounds. This finding suggests that the *n*-BuOH extract and compounds **1–4** and **6** are the best candidates to combat diseases associated with oxidative stress. In accordance with the results of this study, formononetin showed IC_50_ values of 4.65, 9.48, 4.75, 5.0, and 4.27 *μ*g/ml against DPPH, hydroxyl, hydrogen peroxide, nitric oxide, and superoxide radicals, respectively [43]. In addition, previous research has shown that luteolin had strong reactive oxygen species (ROS) scavenging effect against hydroxyl and superoxide radicals compared with that in the control [44]. The results of the present investigation are consistent since ROS generated from activated neutrophils and macrophages have been reported to play an important role in the pathogenesis of various diseases, including neurodegenerative disorders, cancer, and atherosclerosis [45]. This is very promising in terms of discovering antioxidants from plants. Previous studies have illustrated that phenolic compounds including flavonoids and their glycosides are associated with strong antioxidant activity and they possess health benefits [22, 46]. Finally, the phytoconstituents as well as antioxidant/antimicrobial properties of extracts and isolated compounds from *Trifolium baccarinii* are now well established in this study. These results clearly justify the uses of *Trifolium baccarinii* in the treatment of various infectious diseases caused by the tested microorganisms and other ailments associated with oxidative stress.

The results on the bacteriolytic activity showed a decrease in the optical density of bacterial suspensions treated with the MeOH extract and compound **5** as a function of time, indicating the lysis of bacterial cells. Interestingly, none of the tested extracts and compounds showed cytotoxic activity against normal cells. These findings highlight the selective toxicity of the tested samples towards the tested microorganisms. Previous studies have demonstrated some antimicrobial agents causing gross membrane damage [9, 47], and this has been reported previously for flavonoid glycosides from *Graptophyllum grandulosum* [9] and for essential oils from rosewood, oregano, and thyme [47]. The effect of the MeOH extract and compound **5** to lyse *Pseudomonas aeruginosa* and *Staphylococcus aureus* cells suggests that their primary mechanism of action is gross cell wall damage.

The microbial cell membrane permeability and ability to exclude toxic materials or to osmoregulate the cell efficiently may be altered by sublethal injury [48]. As a result, the loss of tolerance to salts or other potentially toxic compounds may be exploited to reveal membrane damage [49] in sublethally injured bacteria. Treatment of *Staphylococcus aureus* with different concentrations of the MeOH extract/compound **5** significantly reduced the number of colony-forming units on media containing NaCl. This effect was most remarkable on culture medium supplemented with 70% NaCl with two times the MICs. These results correlate well with the bacteriolysis results since, in each case, treatment with the MeOH extract/compound **5** at two times the MICs induced the loss of salt tolerance and bacteriolytic effect. Treatment with the MeOH extract/compound **5** caused reduction of superoxide dismutase (SOD) level in *Staphylococcus aureus* strains. In fact, SOD, which catalyzes the dismutation of superoxide into hydrogen peroxide, is the first line of defense in bacterial cells against reactive oxygen species [50]. *Staphylococcus aureus* was facilitated by two major SODs such as SOD-A and SOD-M; the former is mainly involved in endogenous stress while the latter is induced in exogenous stress [50]. Suppression of SOD activity results in decreased conversion of O_2_^•-^ to H_2_O_2_ and consequently culminating in increased O_2_^•-^ levels and leads to oxidative stress-mediated toxicity in *Staphylococcus aureus* cells. Early reports have demonstrated decreases in SOD activity in *Escherichia coli* and *Staphylococcus aureus* upon treatment with the methanolic extract of *Andrographis paniculata*, 2-phenylethynylbutyltellurium (PEBT), and catechin [51–53].

Treatment with the MeOH extract/compound **5** also induced depletion of catalase activity in *Staphylococcus aureus* cell suspension. Only one type of catalase (Kat-A) was found in *Staphylococcus aureus* which is involved in the detoxification of H_2_O_2_ by converting it into H_2_O and O_2_ with the help of a heme cofactor [54]. Hence, decrease in catalase level caused by the MeOH extract/compound **5** might result in increased H_2_O_2_ level and leads to oxidative stress-mediated toxicity in *Staphylococcus aureus* cell suspension. Identical to our findings, the *Leonurus cardiaca* extract and other phytocompounds like catechin, allylpyrocatechol, and silibin also lowered the catalase activity and provoked toxicity in *Staphylococcus aureus* [53, 55–57]. Altogether, these results suggest that the MeOH extract and compound **5** are promising lead candidates with antibacterial potential against MRSA. Thus, these samples exhibited antibacterial activity through the bacteriolytic effect and reduction of the antioxidant defenses in the bacterial cells.

## 5. Conclusion

Given the results obtained in the present study, we can conclude that the purification of the EtOAc and *n*-BuOH extracts from *Trifolium baccarinii* led to the isolation and characterization of ten known compounds, namely, biochanin A (**1**), formononetin (**2**), luteolin (**3**), luteolin-4′-O-*β*-D-glucopyranoside (**4**), 4,7,2′-trihydroxy-4′-methoxyisoflavanol (**5**), sissotrin (**6**), 1-methyl-*β*-D-glucopyranoside (**7**), ononin (**8**), D-mannitol (**9**), and 3-O-*β*-D-glucuronopyranosylsoyasapogenol B (**10**). The MeOH, EtOAc, and *n*-BuOH extracts as well as compounds **1**–**6** from *Trifolium baccarinii* displayed the most antimicrobial and antioxidant activities. The MeOH extract and compound **5** exhibited antibacterial activity through bacteriolytic effects and reduction of the antioxidant defenses in the bacterial cells. This is the first report on the mechanisms of antibacterial action of the MeOH extract and compound **5** from *Trifolium baccarinii* against pathogenic strains. None of the tested extracts/compounds showed cytotoxic activity against normal cells, highlighting their high selectivity toward pathogenic bacteria and yeasts. The MeOH extract and compound **5** are promising lead candidates with antibacterial potential against methicillin-resistant *Staphylococcus aureus*. Hence, they can be utilized to fight against infectious diseases caused by the tested microorganisms and to combat diseases that induce oxidative stress.

## Figures and Tables

**Table 1:** Antimicrobial activity (MIC and MMC in *μ*g/mL) of extracts and isolated compounds from *Trifolium baccarinii* as well as reference antimicrobial drugs.

Extracts/compounds	Inhibition parameters	*Pseudomonas aeruginosa* ATCC27853	*Escherichia coli* S2(1)	*Staphylococcus aureus* ATCC25923	MRSA03	MRSA04	*Candida albicans* ATCC10231	*Cryptococcus neoformans* H99
MeOH extract	MIC	32	64	32	32	64	64	32
	MMC	64	128	32	64	64	128	64
	MMC/MIC	2	2	1	2	1	2	2

EtOAc extract	MIC	64	64	32	32	32	64	32
	MMC	128	128	64	64	64	256	128
	MMC/MIC	2	2	2	2	2	4	4

*n*-BuOH extract	MIC	256	128	256	128	512	128	128
	MMC	512	1024	512	512	2048	256	128
	MMC/MIC	2	8	2	4	4	2	1

**1**	MIC	32	32	32	64	64	32	32
	MMC	64	64	32	128	128	64	32
	MMC/MIC	2	2	1	2	2	2	1

**2**	MIC	128	64	64	128	128	64	64
	MMC	128	128	128	256	256	128	64
	MMC/MIC	1	2	2	2	2	2	1

**3**	MIC	32	32	32	16	32	16	8
	MMC	64	32	64	16	64	32	16
	MMC/MIC	2	1	2	1	2	2	2

**4**	MIC	64	32	32	64	64	16	16
	MMC	128	32	64	128	128	16	16
	MMC/MIC	2	1	2	2	2	1	1

**5**	MIC	8	8	8	16	16	8	8
	MMC	8	16	8	32	16	16	8
	MMC/MIC	1	2	1	2	1	2	1

**6**	MIC	128	64	64	256	256	64	32
	MMC	256	128	128	>256	>256	>256	256
	MMC/MIC	2	2	2	/	/	/	8

**7**	MIC	>256	>256	>256	>256	>256	>256	>256
	MMC	>256	>256	>256	>256	>256	>256	>256
	MMC/MIC	/	/	/	/	/	/	/

**8**	MIC	256	128	64	256	256	128	64
	MMC	256	256	256	>256	>256	>256	>256
	MMC/MIC	1	2	4	/	/	/	/

**9**	MIC	256	128	128	>256	>256	>256	256
	MMC	>256	>256	>256	>256	>256	>256	>256
	MMC/MIC	/	/	/	/	/	/	/

**10**	MIC	256	256	256	>256	>256	>256	256
	MMC	>256	>256	>256	>256	>256	>256	>256
	MMC/MIC	/	/	/	/	/	/	/

Ref^∗^	MIC	2	16	1	4	8	1	2
	MMC	2	32	1	8	8	1	2
	MMC/MIC	1	2	1	2	1	1	1

/: not determined; MIC: minimum inhibitory concentration; MMC: minimum microbicidal concentration; ∗: nystatin for yeasts and oxacillin for bacteria; MRSA03: methicillin-resistant *Staphylococcus aureus* 03; MRSA04: methicillin-resistant *Staphylococcus aureus* 04.

**Table 2:** Antioxidant activities (EC_50_ and GEAC in *μ*g/mL) of extracts and some isolated compounds from *Trifolium baccarinii*.

Extracts/compounds	DPPH free radical scavenging activity (EC_50_)	Gallic acid equivalent antioxidant capacity (GEAC)
MeOH extract	98.26 ± 0.74^a^	35.59 ± 0.63^a^
EtOAc extract	105.13 ± 0.86^b^	27.43 ± 1.01^b^
*n*-BuOH extract	81.74 ± 0.98^c^	46.14 ± 1.26^c^
**1**	4.87 ± 0.43^d^	101.77 ± 1.24^d^
**2**	6.39 ± 0.62^e^	77.91 ± 0.59^e^
**3**	8.02 ± 1.16^e^	68.30 ± 0.71^f^
**4**	3.71 ± 0.99^d^	118.71 ± 0.19^g^
**5**	11.58 ± 0.73^f^	61.05 ± 0.90^h^
**6**	7.13 ± 0.97^e^	72.96 ± 1.54^i^
**8**	38.96 ± 1.54^g^	57.43 ± 0.97^j^
Vitamin C	1.81 ± 0.19^h^	/

EC_50_: equivalent concentrations of test samples scavenging 50% of DPPH radical; /: not determined. Data represent the mean ± SD of three independent experiments carried out in triplicate. In the same column, values earmarked by different superscript letters (a–j) are significantly different according to one-way ANOVA and the Waller-Duncan test (*p* < 0.05).

## Data Availability

The datasets generated and analyzed during the current study are available from the corresponding author upon reasonable request.

## Abbreviations

^13^C-NMR: Carbon thirteen nuclear magnetic resonance
^1^H-NMR: Proton nuclear magnetic resonance
2D NMR: Two-dimension nuclear magnetic resonance
ATCC: American Type Culture Collection
CC: Column chromatography
COSY: Correlation spectroscopy
DMSO: Dimethyl sulfoxide
EtOAc: Ethyl acetate
HMBC: Heteronuclear multiple bond connectivities
HNC: Herbier National du Cameroun
HSQC: The heteronuclear single quantum coherence
IR: Infrared
MBC: Minimum bactericidal concentration
MDR: Multidrug-resistant
MeOH: Methanol
MHA: Mueller-Hinton agar
MHB: Mueller-Hinton broth
MIC: Minimum inhibitory concentration
MMC: Minimum microbicidal concentration
MRSA03: Methicillin-resistant *Staphylococcus aureus* 03
MRSA04: Methicillin-resistant *Staphylococcus aureus* 04
NA: Nutrient agar
*n*-BuOH: *n*-Butanol
NMR: Nuclear magnetic resonance
Rf: Retention factor
TLC: Thin-layer chromatography
TMS: Tetramethylsilane
UV: Ultraviolet.

## Supplementary Materials

Supplementary Materials Figure S1: ^1^H NMR spectrum of compound **1**. Figure S2: ^13^C NMR spectrum of compound **1**. Figure S3: ^1^H-NMR spectrum of compound **2**. Figure S4: ^13^C-NMR spectrum of compound **2**. Figure S5: HSQC spectrum of compound **2**. Figure S6: ^1^H-^1^H COSY spectrum of compound **2**. Figure S7: HMBC spectrum of compound **2**. Figure S8: ^1^H-NMR spectrum of compound **3**. Figure S9: ^13^C-NMR spectrum of compound **3**. Figure S10: HSQC spectrum of compound **3**. Figure S11: ^1^H-NMR spectrum of compound **4**. Figure S12: ^13^C-NMR spectrum of compound **4**. Figure S13: HSQC spectrum of compound **4**. Figure S14: ^1^H-^1^H COSY spectrum of compound **4**. Figure S15: HMBC spectrum of compound **4**. Figure S16: ^1^H-NMR spectrum of compound **5**. Figure S17: ^13^C-NMR spectrum of compound **5**. Figure S18: HSQC spectrum of compound **5**. Figure S19: ^1^H-^1^H COSY spectrum of compound **5**. Figure S20: HMBC spectrum of compound **5**. Figure S21: ^1^H-NMR spectrum of compound **6**. Figure S22: ^13^C-NMR spectrum of compound **6**. Figure S23: HSQC spectrum of compound **6**. Figure S24: ^1^H-^1^H COSY spectrum of compound **6**. Figure S25: HMBC spectrum of compound **6**. Figure S26: ^1^H-NMR spectrum of compound **7**. Figure S27: ^13^C-NMR spectrum of compound **7**. Figure S28: HSQC spectrum of compound **7**. Figure S29: ^1^H-^1^H COSY spectrum of compound **7**. Figure S30: HMBC spectrum of compound **7**. Figure S31: ^1^H-NMR spectrum of compound **8**. Figure S32: ^13^C-NMR spectrum of compound **8**. Figure S33: HSQC spectrum of compound **8**. Figure S34: ^1^H-^1^H COSY spectrum of compound **8**. Figure S35: HMBC spectrum of compound **8**. Figure S36: ^1^H-NMR spectrum of compound **9**. Figure S37: ^13^C-NMR spectrum of compound **9**. Figure S38: HSQC spectrum of compound **9**. Figure S39: ^1^H-NMR spectrum of compound **10**. Figure S40: ^13^C-NMR spectrum of compound **10**. Figure S41: HSQC spectrum of compound **10**. Figure S42: ^1^H-^1^H COSY spectrum of compound **10**. Figure S43: HMBC spectrum of compound **10** (supplementary materials/figures).
