# Supplementary material for: Antimicrobial and Antioxidant Secondary Metabolites from Trifolium baccarinii Chiov. (Fabaceae) and Their Mechanisms of Antibacterial Action
Source: Biomed Res Int. 2021 Oct 22;2021:3099428. doi: 10.1155/2021/3099428 (PMC8556085; doi:10.1155/2021/3099428)
Supplement: Supplementary Materials — Figure S1: 1H NMR spectrum of compound 1. Figure S2: 13C NMR spectrum of compound 1. Figure S3: 1H-NMR spectrum of compound 2. Figure S4: 13C-NMR spectrum of compound 2. Figure S5: HSQC spectrum of compound 2. Figure S6: 1H-1H COSY spectrum of compound 2. Figure S7: HMBC spectrum of compound 2. Figure S8: 1H-NMR spectrum of compound 3. Figure S9: 13C-NMR spectrum of compound 3. Figure S10: HSQC spectrum of compound 3. Figure S11: 1H-NMR spectrum of compound 4. Figure S12: 13C-NMR spectrum of compound 4. Figure S13: HSQC spectrum of compound 4. Figure S14: 1H-1H COSY spectrum of compound 4. Figure S15: HMBC spectrum of compound 4. Figure S16: 1H-NMR spectrum of compound 5. Figure S17: 13C-NMR spectrum of compound 5. Figure S18: HSQC spectrum of compound 5. Figure S19: 1H-1H COSY spectrum of compound 5. Figure S20: HMBC spectrum of compound 5. Figure S21: 1H-NMR spectrum of compound 6. Figure S22: 13C-NMR spectrum of compound 6. Figure S23: HSQC spectrum of compound 6. Figure S24: 1H-1H COSY spectrum of compound 6. Figure S25: HMBC spectrum of compound 6. Figure S26: 1H-NMR spectrum of compound 7. Figure S27: 13C-NMR spectrum of compound 7. Figure S28: HSQC spectrum of compound 7. Figure S29: 1H-1H COSY spectrum of compound 7. Figure S30: HMBC spectrum of compound 7. Figure S31: 1H-NMR spectrum of compound 8. Figure S32: 13C-NMR spectrum of compound 8. Figure S33: HSQC spectrum of compound 8. Figure S34: 1H-1H COSY spectrum of compound 8. Figure S35: HMBC spectrum of compound 8. Figure S36: 1H-NMR spectrum of compound 9. Figure S37: 13C-NMR spectrum of compound 9. Figure S38: HSQC spectrum of compound 9. Figure S39: 1H-NMR spectrum of compound 10. Figure S40: 13C-NMR spectrum of compound 10. Figure S41: HSQC spectrum of compound 10. Figure S42: 1H-1H COSY spectrum of compound 10. Figure S43: HMBC spectrum of compound 10 (supplementary materials/figures). [file 3099428.f1.pdf]

Antimicrobial and antioxidant secondary metabolites from *Trifolium baccarinii* Chiov. (Fabaceae) and their mechanisms of antibacterial action

Léonel Donald Tsamo Feugap[1], Jean-De-Dieu Tamokou[2,*], Irene Chinda Kengne[2], Claudia Darille Jouogo Ngnokam[1], Mahamat Djamalladine Djamalladine[1], Laurence Voutquenne-Nazabadioko[3] and David Ngnokam[1]

Supplementary Material

FIGURE captions

Figure S1: ^{1}H NMR Spectrum of Compound **1** 4

FIGURE S2: ^{13}C NMR Spectrum of Compound **1** 4

FIGURE S3: ^{1}H-NMR Spectrum of Compound **2** 4

FIGURE S4: ^{13}C-NMR Spectrum of Compound **2** 5

FIGURE S5: HSQC Spectrum of Compound **2** 5

FIGURE S6 : ^{1}H-^{1}H COSY Spectrum of Compound **2** 6

FIGURE S7: HMBC Spectrum of Compound **2** 6

FIGURE S8 : ^{1}H-NMR Spectrum of Compound **3** 7

FIGURE S9 : ^{13}C-NMR Spectrum of Compound **3** 7

FIGURE S10 : HSQC Spectrum of Compound **3** 8

FIGURE S11 : ^{1}H-NMR Spectrum of Compound **4** 8

FIGURE S12 : ^{13}C-NMR Spectrum of Compound **4** 9

FIGURE S13 : HSQC Spectrum of Compound **4** 9

FIGURE S14 : ^{1}H-^{1}H COSY Spectrum of Compound **4** 10

FIGURE S15 : HMBC Spectrum of Compound **4** 10

FIGURE S16 : ^{1}H-NMR Spectrum of Compound **5** 11

FIGURE S17 : ^{13}C-NMR Spectrum of Compound **5** 11

FIGURE S18 : HSQC Spectrum of Compound **5** 12

FIGURE S19 : ^{1}H-^{1}H COSY Spectrum of Compound **5** 12

FIGURE S20 : HMBC Spectrum of Compound **5** 13

FIGURE S21 : ^{1}H-NMR Spectrum of Compound **6** 13

FIGURE S22 : ^{13}C-NMR Spectrum of Compound **6** 13

FIGURE S23 : HSQC Spectrum of Compound **6** 14

FIGURE S24 : ^{1}H-^{1}H COSY Spectrum of Compound **6** 14

FIGURE S25 : HMBC Spectrum of Compound **6** 15

FIGURE S26 : ^{1}H-NMR Spectrum of Compound **7** 15

FIGURE S27 : ^{13}C-NMR Spectrum of Compound **7** 16

FIGURE S28 : HSQC Spectrum of Compound **7** 16

FIGURE S29 : ^{1}H-^{1}H COSY Spectrum of Compound **7** 17

FIGURE S30 : HMBC Spectrum of Compound **7** 17

FIGURE S31 : ^{1}H-NMR Spectrum of Compound **8** 18

FIGURE S32 : ^{13}C-NMR Spectrum of Compound **8** 18

FIGURE S33 : HSQC Spectrum of Compound **8** 19

FIGURE S34 : ^{1}H-^{1}H COSY Spectrum of Compound **8** 19

FIGURE S35 : HMBC Spectrum of Compound **8** 20

FIGURE S36 : ^{1}H-NMR Spectrum of Compound **9** 20

FIGURE S37 : ^{13}C-NMR Spectrum of Compound **9** 21

FIGURE S38 : HSQC Spectrum of Compound **9** 21

FIGURE S39 : ^{1}H-NMR Spectrum of Compound **10** 22

FIGURE S40 : ^{13}C-NMR Spectrum of Compound **10** 22

FIGURE S41 : HSQC Spectrum of Compound **10** 23

FIGURE S42 : ^{1}H-^{1}H COSY Spectrum of Compound **10** 23

FIGURE S43 : HMBC Spectrum of Compound **10** 24

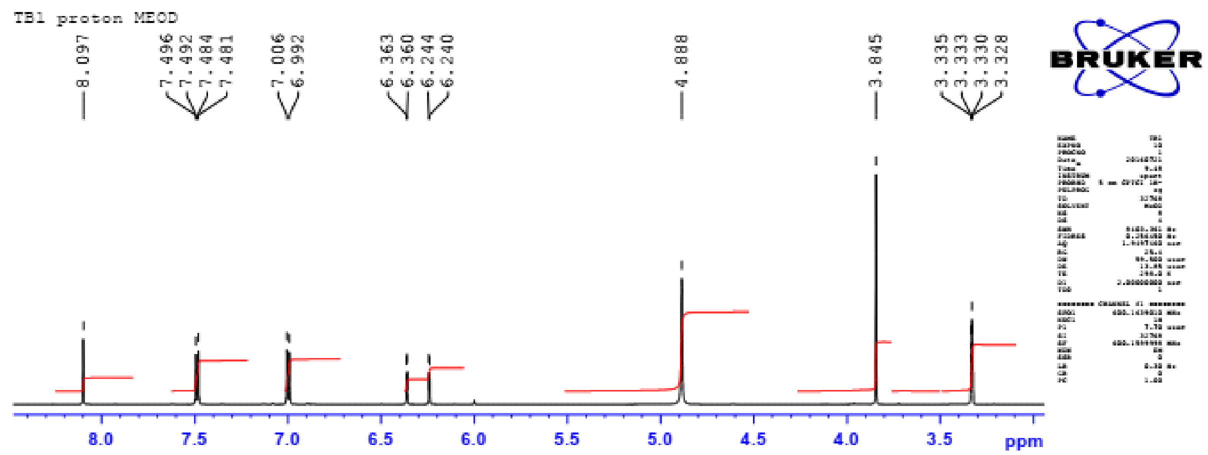

FIGURE S1: ^{1}H NMR Spectrum of Compound **1**

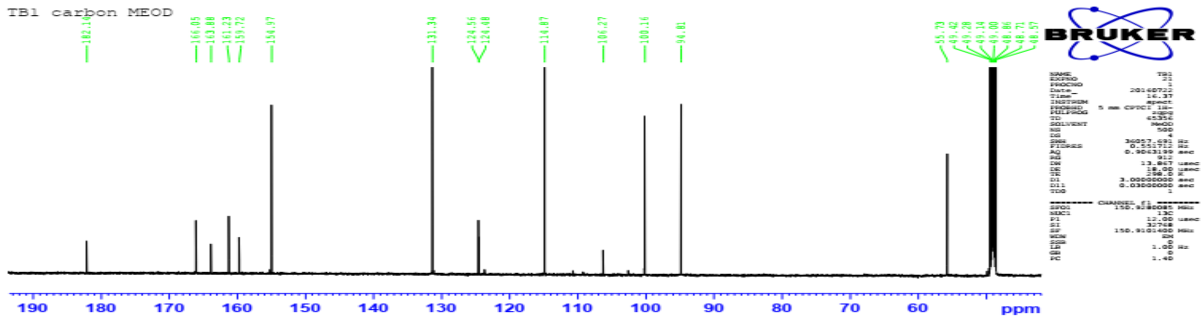

FIGURE S2. ^{13}C NMR Spectrum of Compound **1**

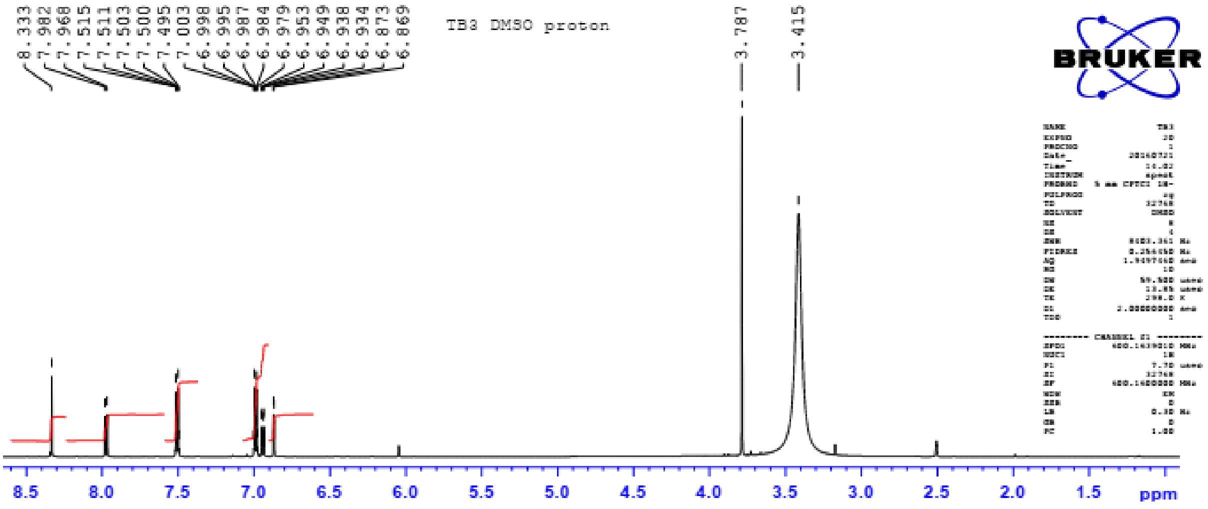

FIGURE S3: ^{1}H-NMR Spectrum of Compound **2**

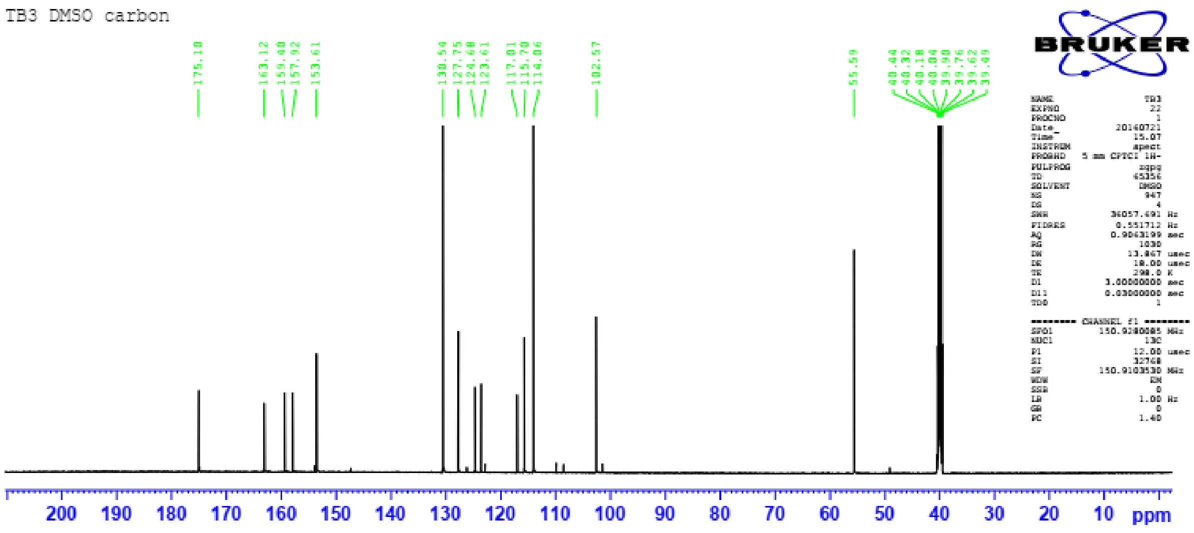

FIGURE S4: ^{13}C-NMR Spectrum of Compound **2**

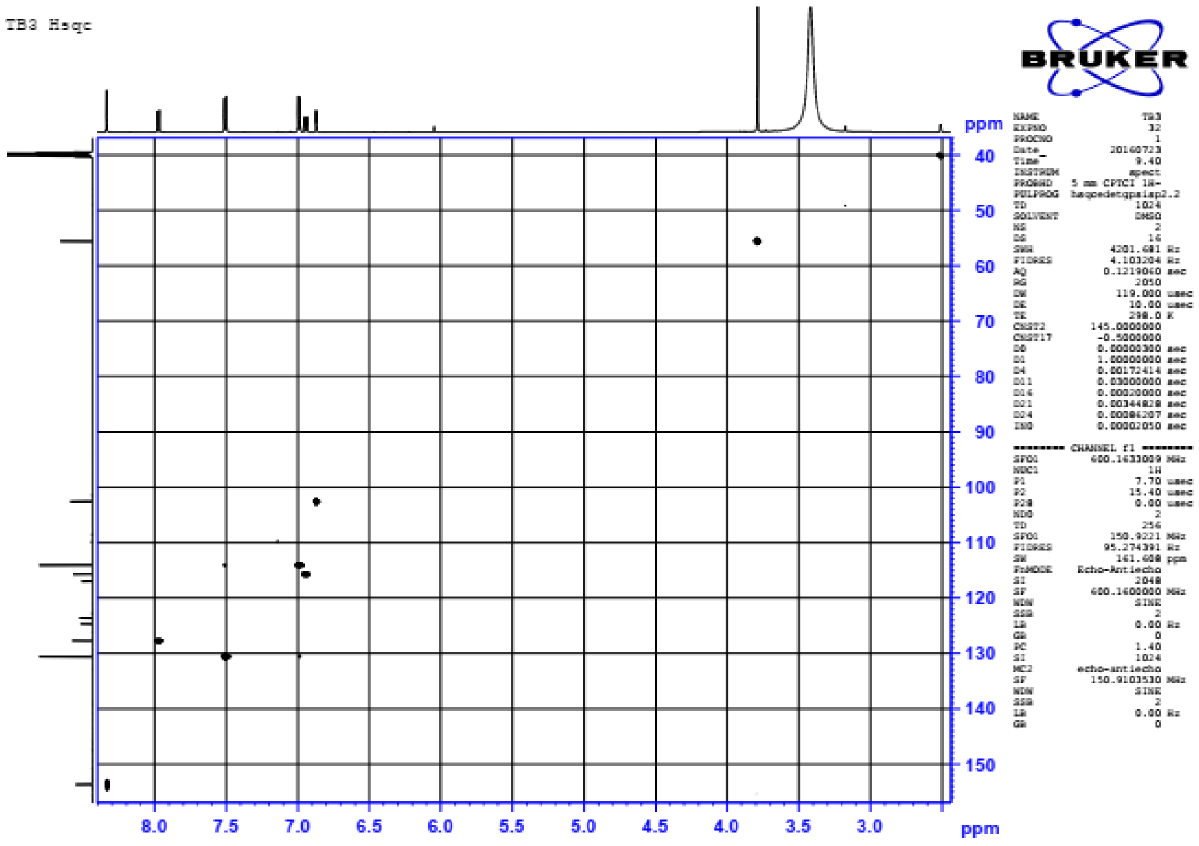

FIGURE S5: HSQC Spectrum of Compound **2**

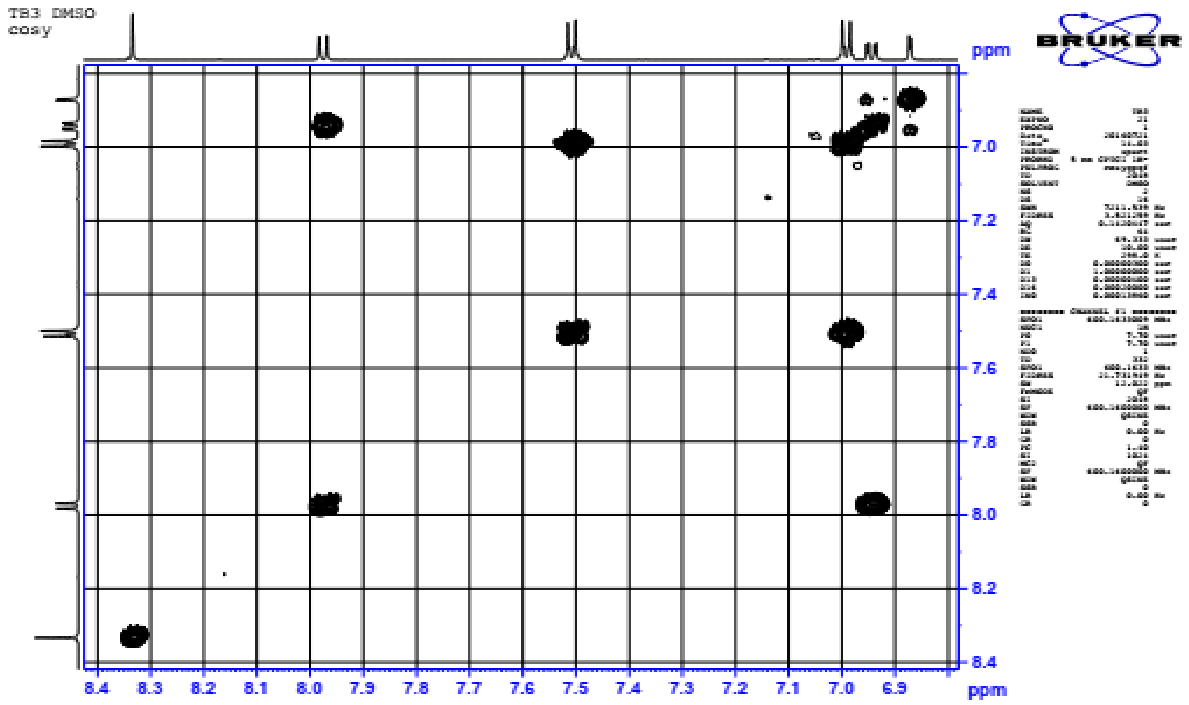

FIGURE S6 : ^{1}H-^{1}H COSY Spectrum of Compound **2**

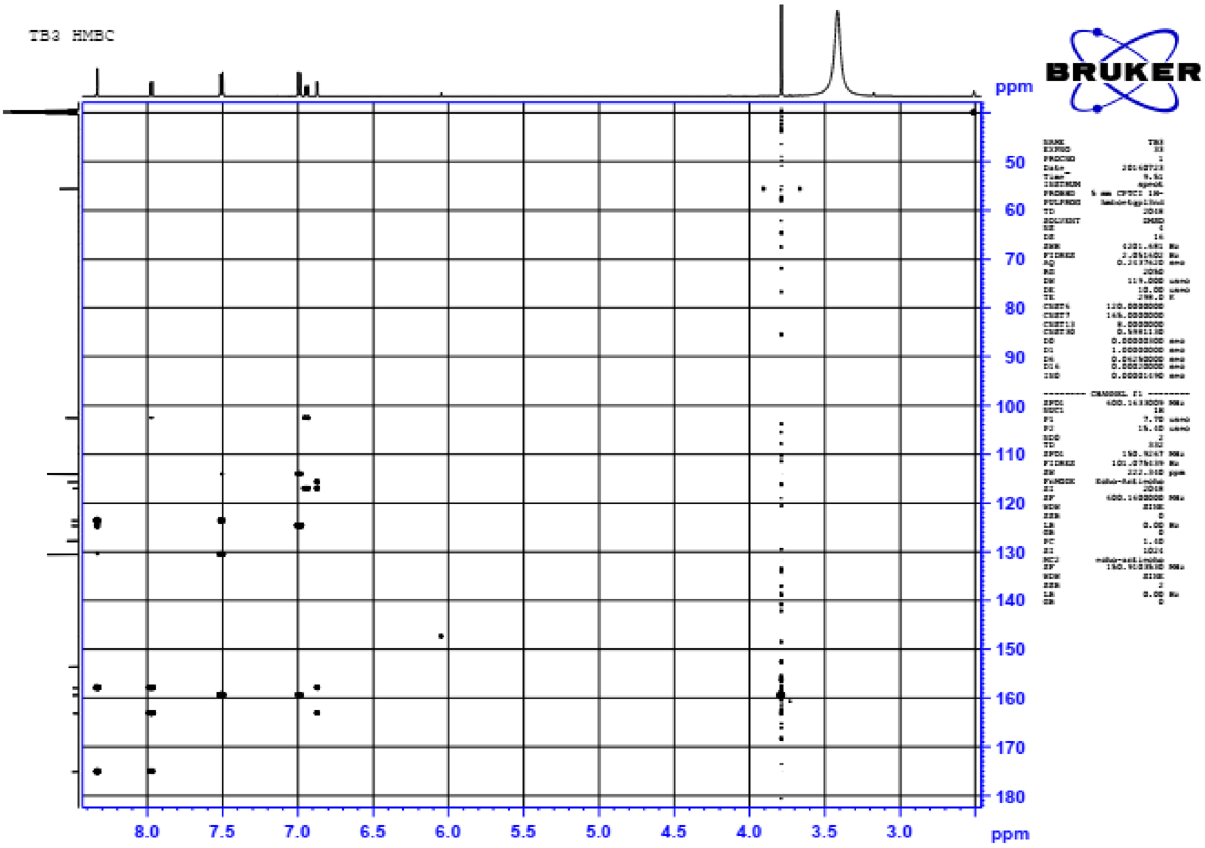

FIGURE S7: HMBC Spectrum of Compound **2**

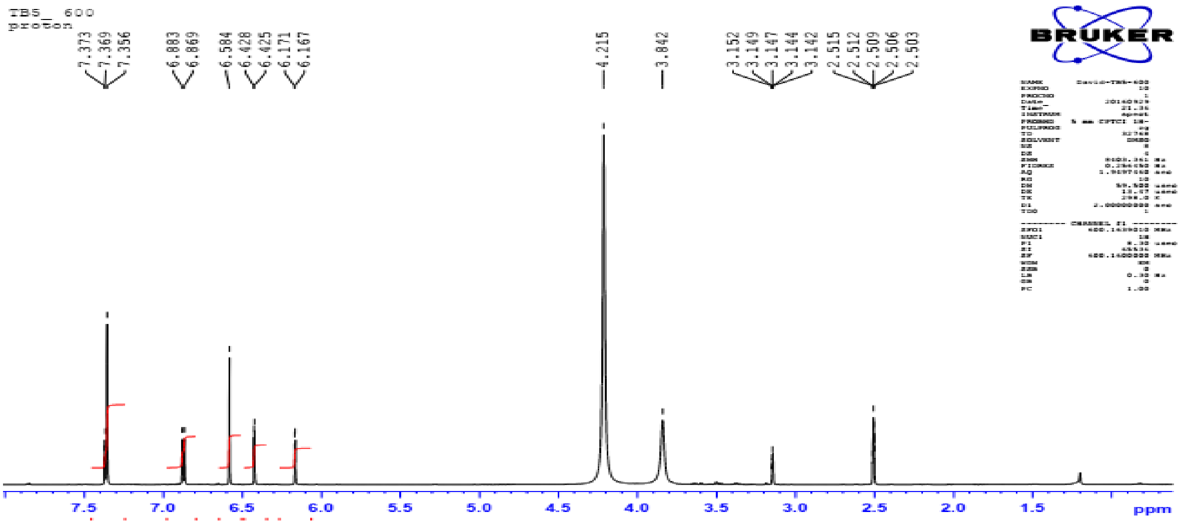

FIGURE S8 : ^{1}H-NMR Spectrum of Compound **3**

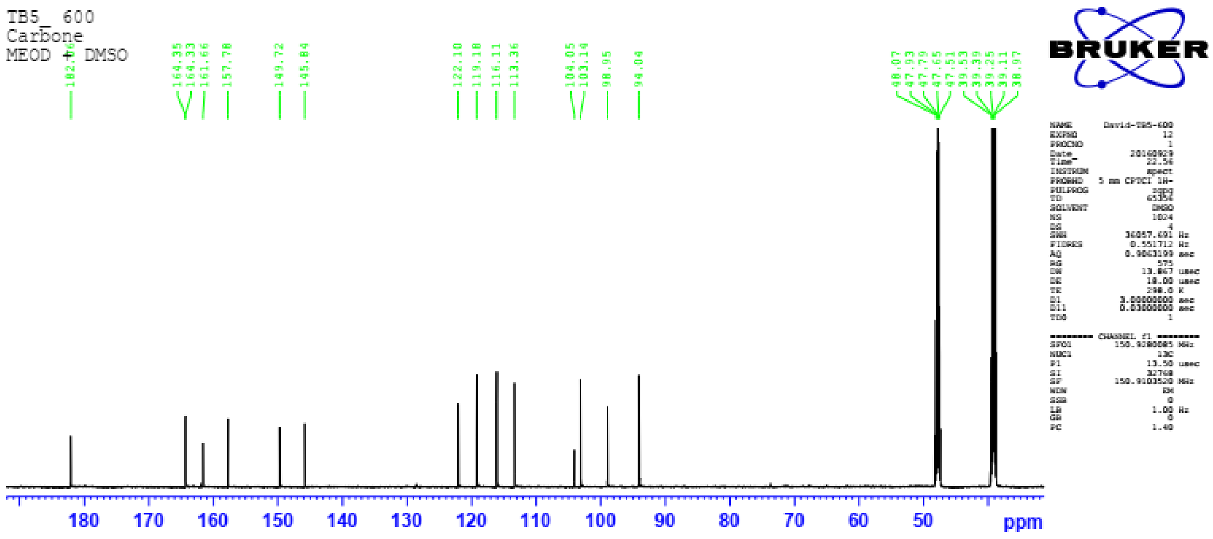

FIGURE S9 : ^{13}C-NMR Spectrum of Compound **3**

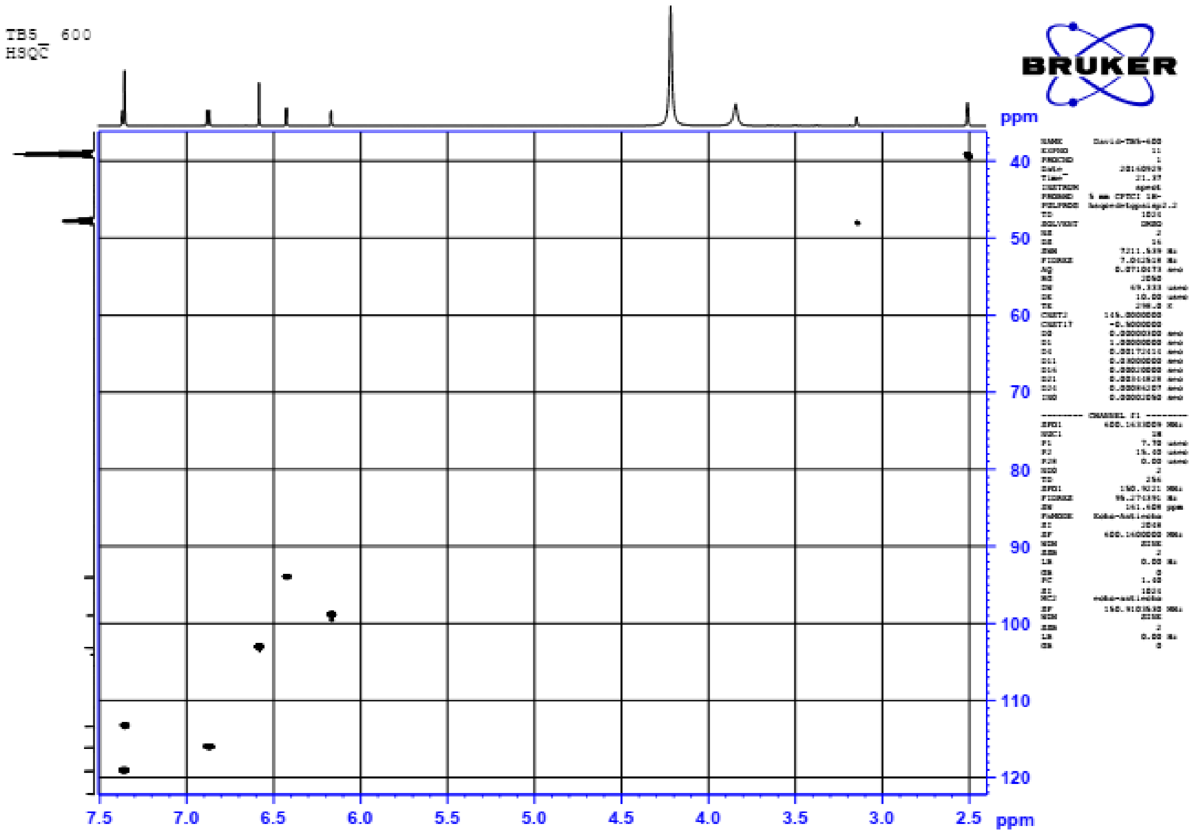

FIGURE S10 : HSQC Spectrum of Compound **3**

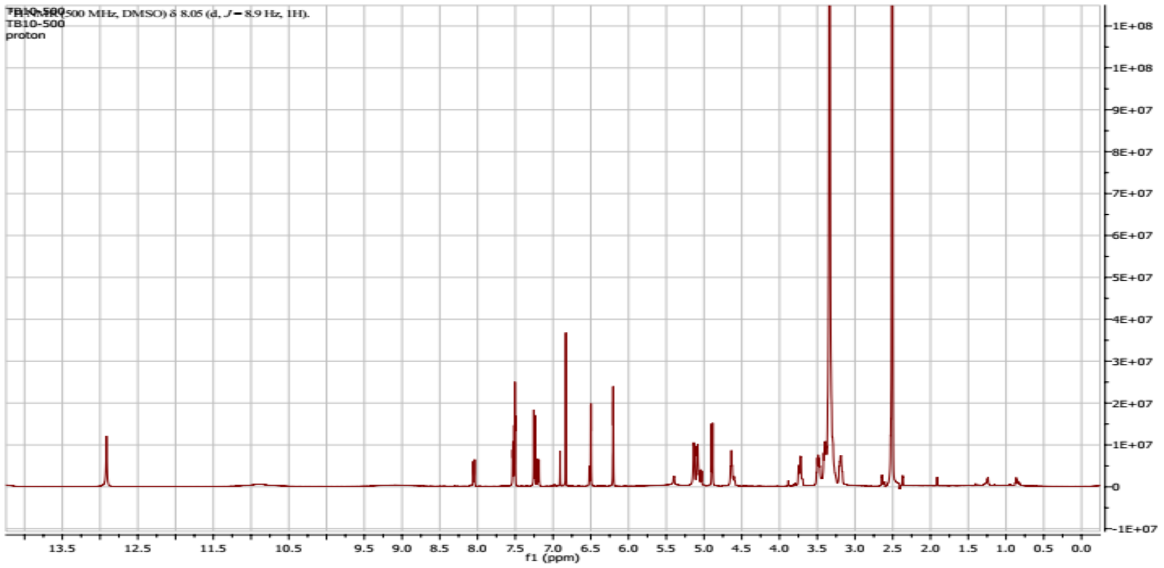

FIGURE S11 : ^{1}H-NMR Spectrum of Compound **4**

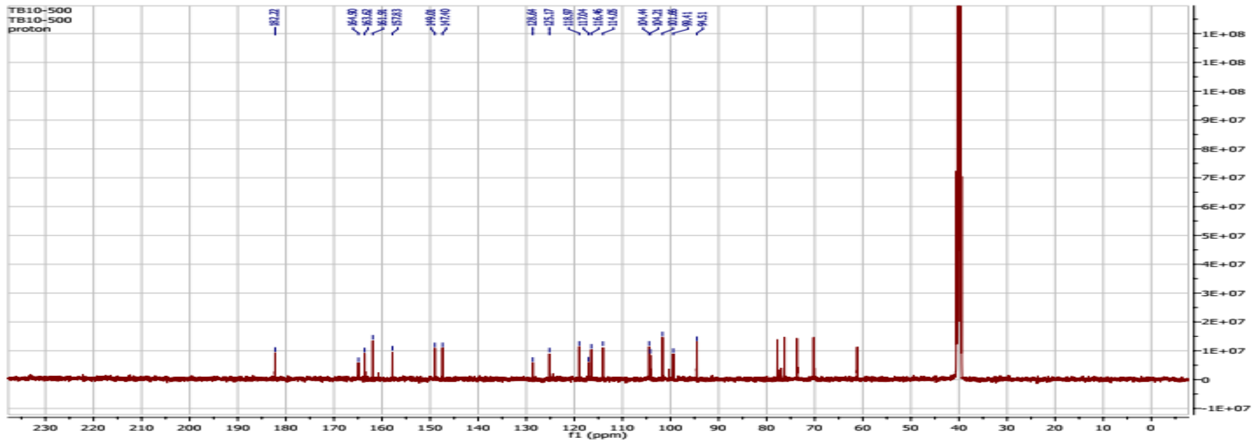

FIGURE S12 : ^{13}C-NMR Spectrum of Compound **4**

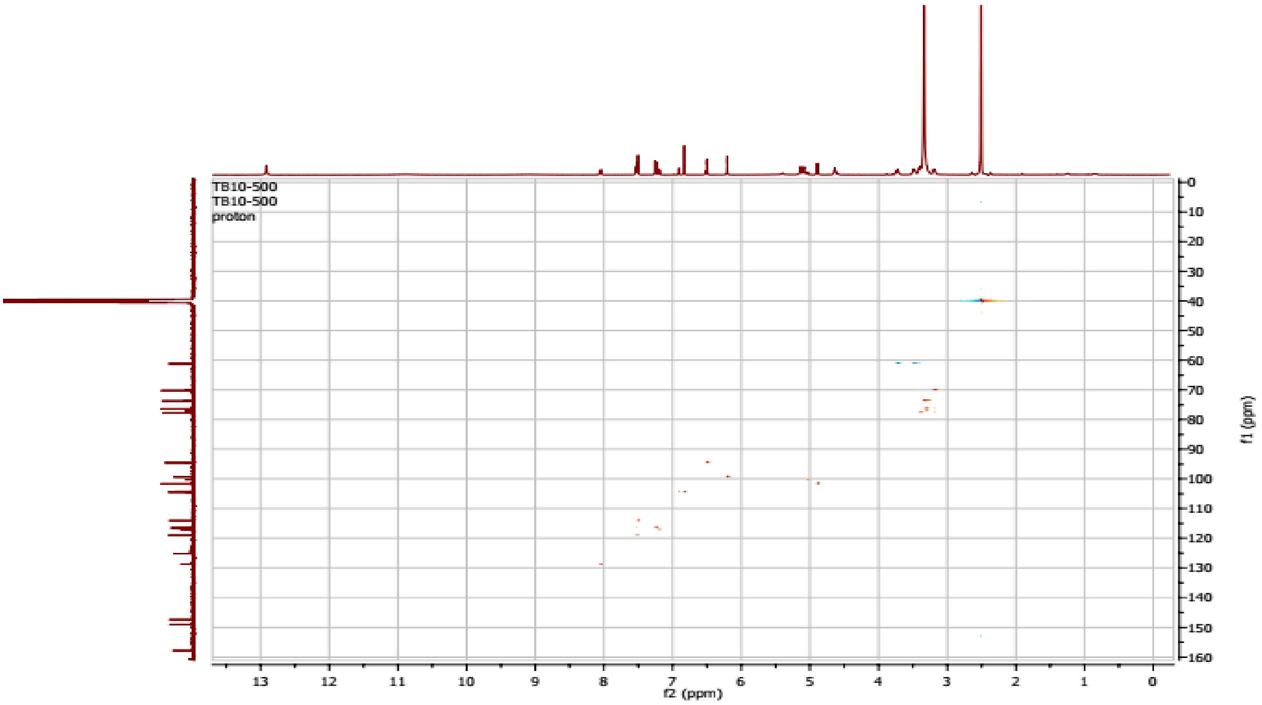

FIGURE S13 : HSQC Spectrum of Compound **4**

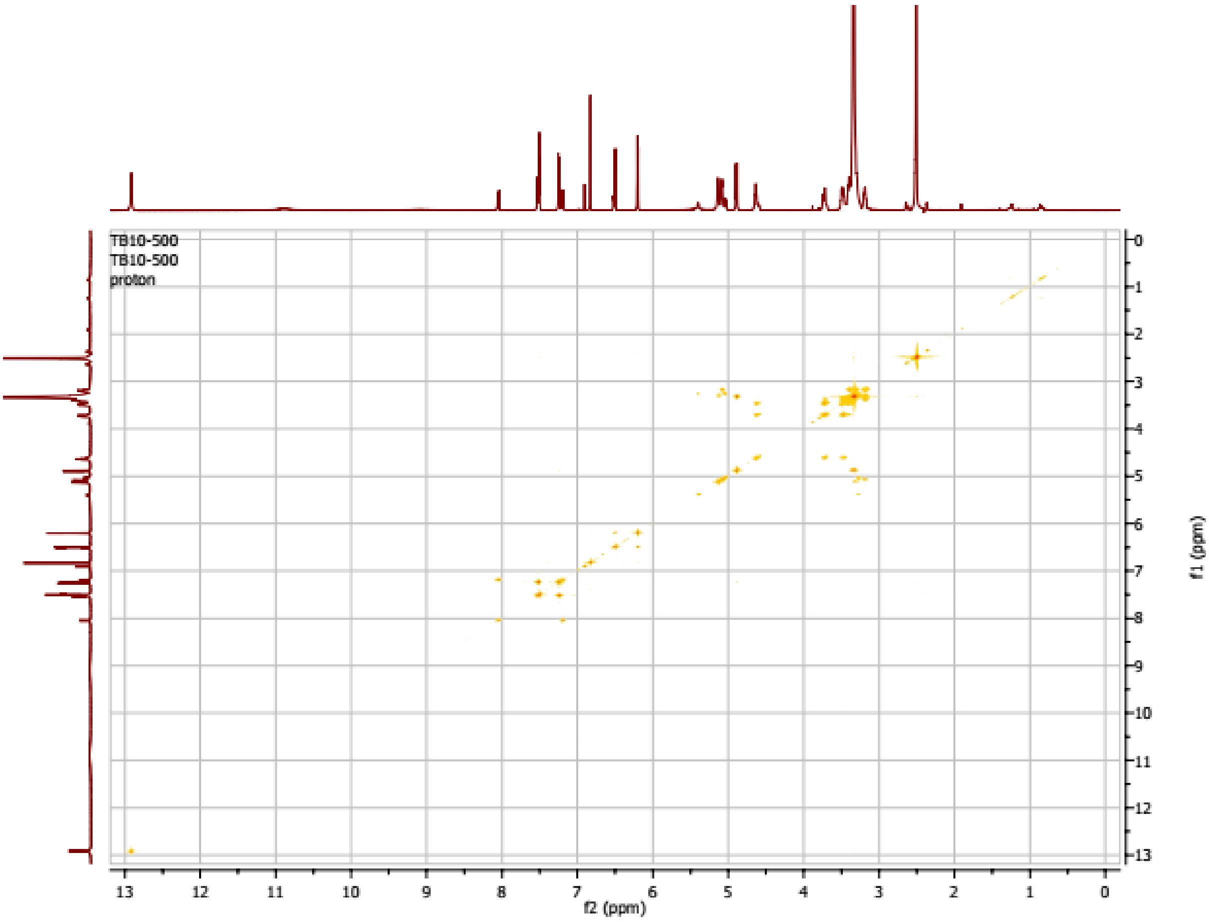

FIGURE S14 : ^{1}H-^{1}H COSY Spectrum of Compound **4**

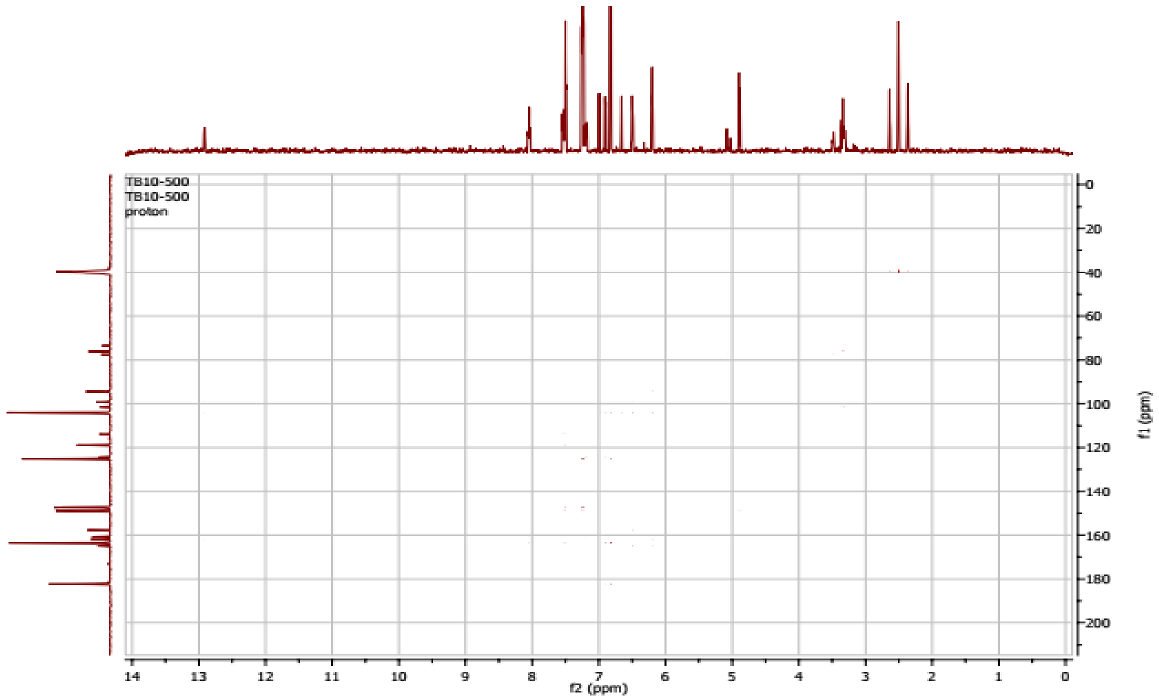

FIGURE S15 : HMBC Spectrum of Compound **4**

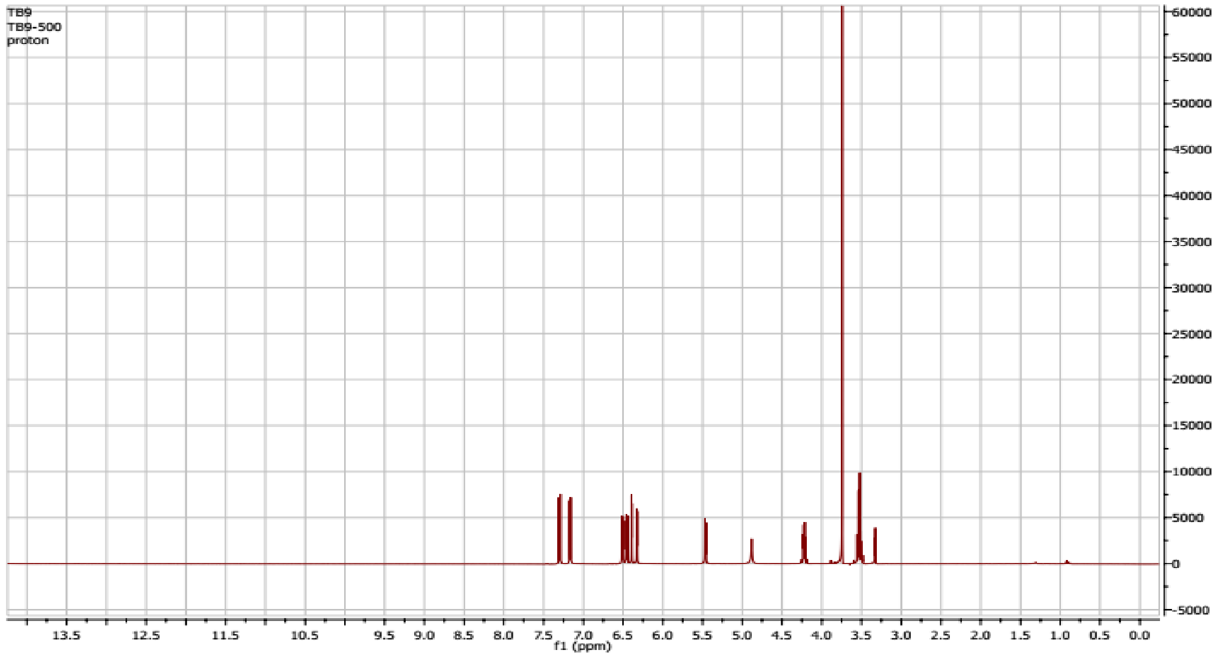

FIGURE S16 : ^{1}H-NMR Spectrum of Compound **5**

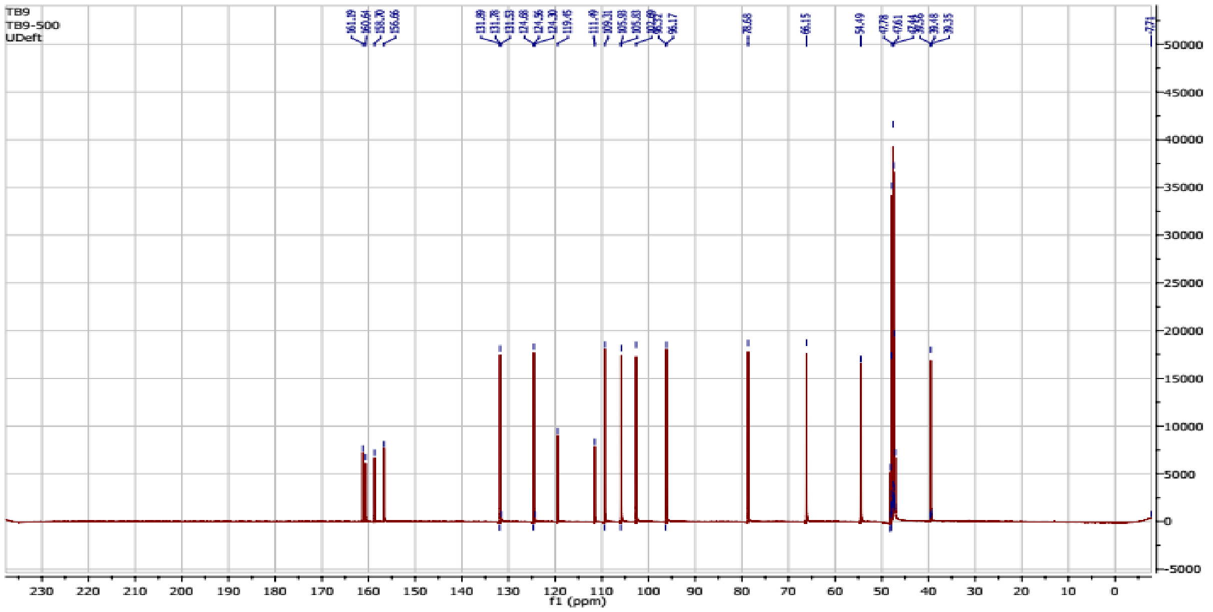

FIGURE S17 : ^{13}C-NMR Spectrum of Compound **5**

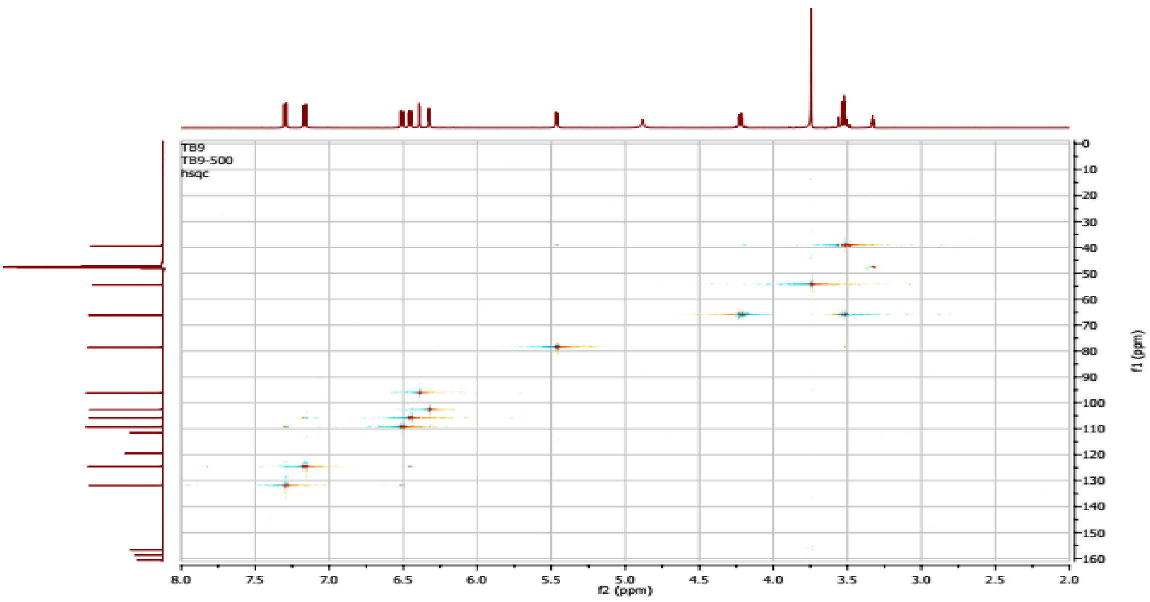

FIGURE S18 : HSQC Spectrum of Compound **5**

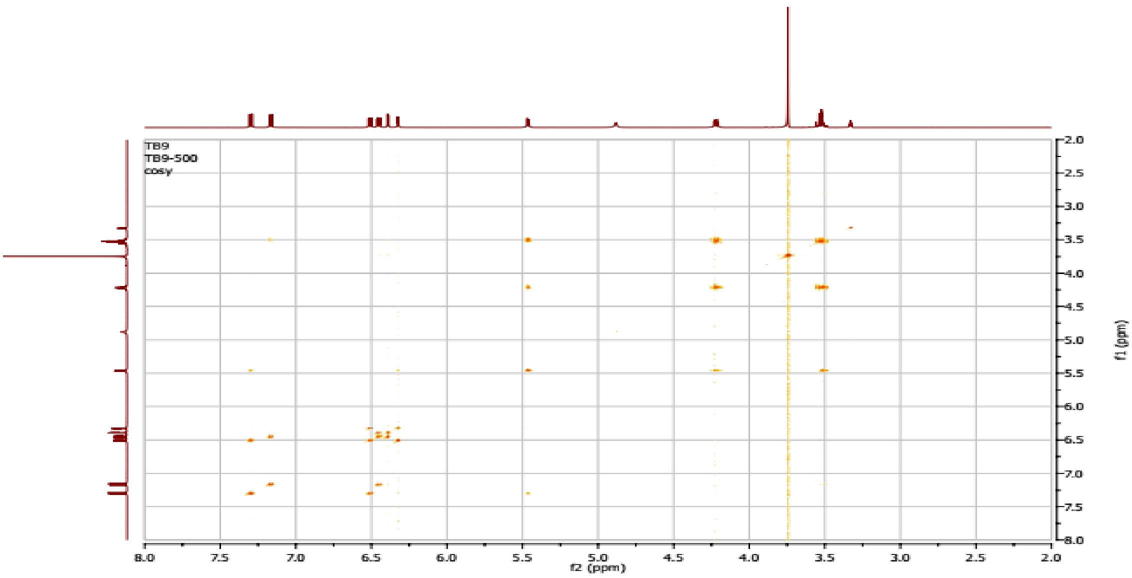

FIGURE S19 : ^{1}H-^{1}H COSY Spectrum of Compound **5**

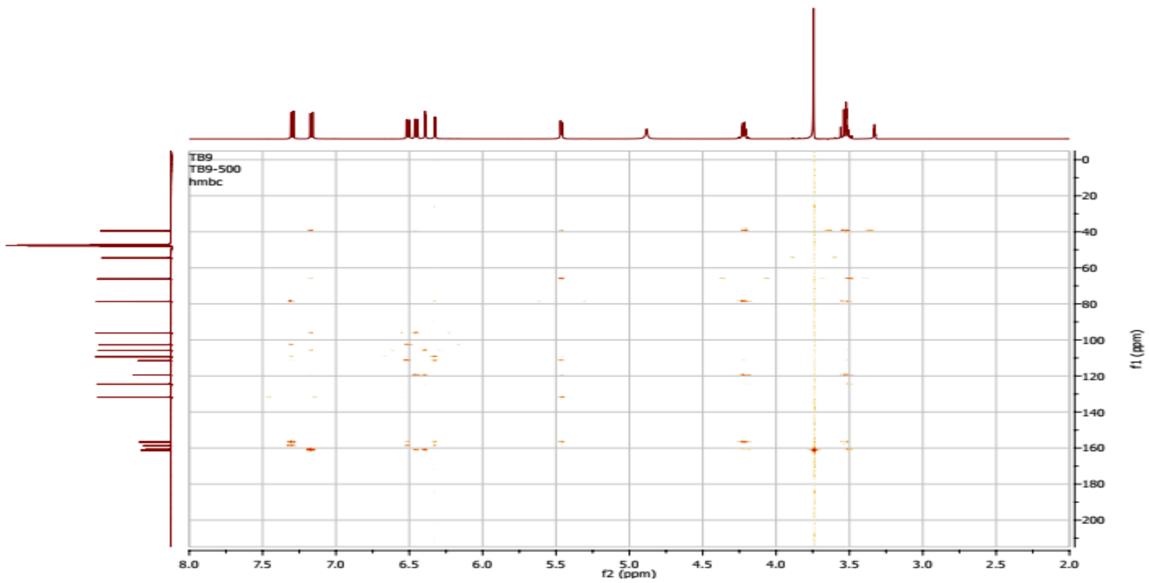

FIGURE S20 : HMBC Spectrum of Compound **5**

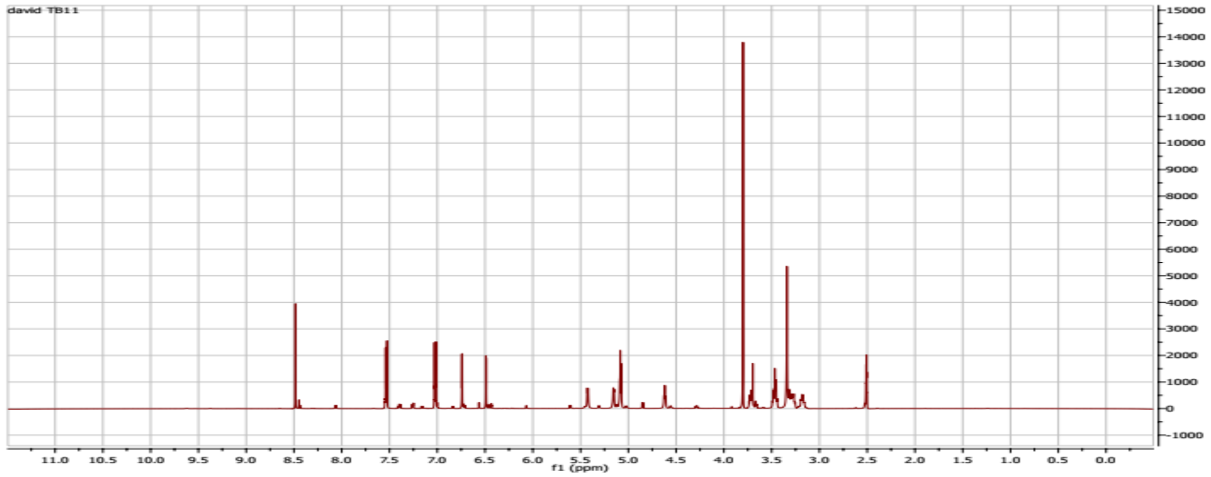

FIGURE S21 : ^{1}H-NMR Spectrum

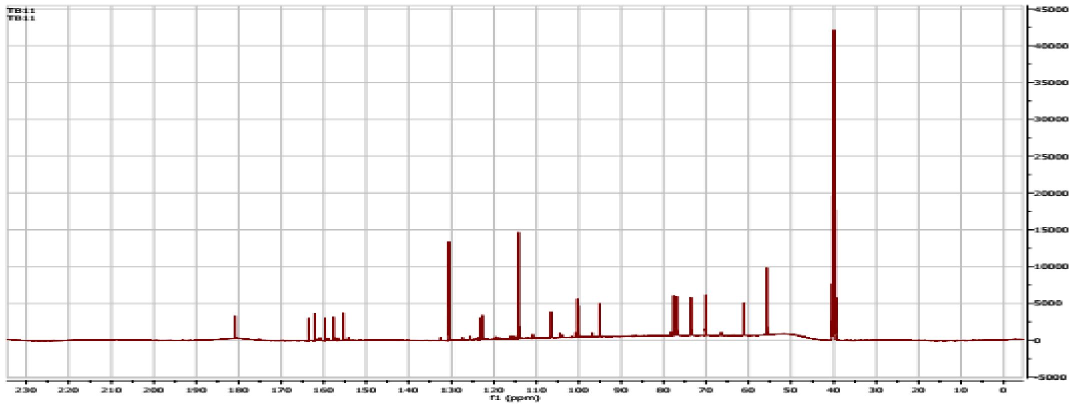

FIGURE S22 : ^{13}C-NMR Spectrum of Compound **6**

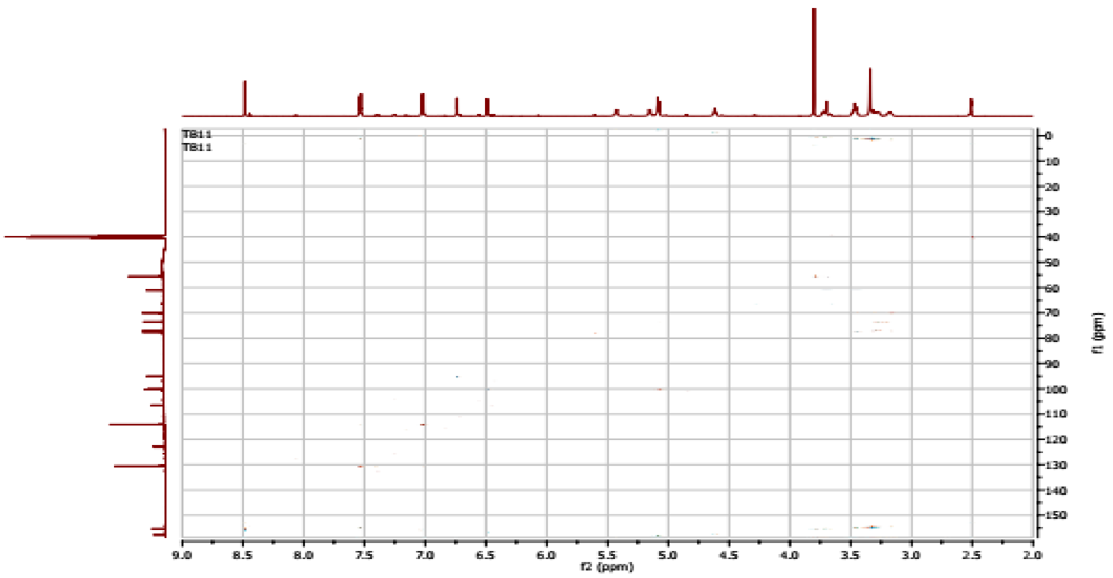

FIGURE S23 : HSQC Spectrum of Compound **6**

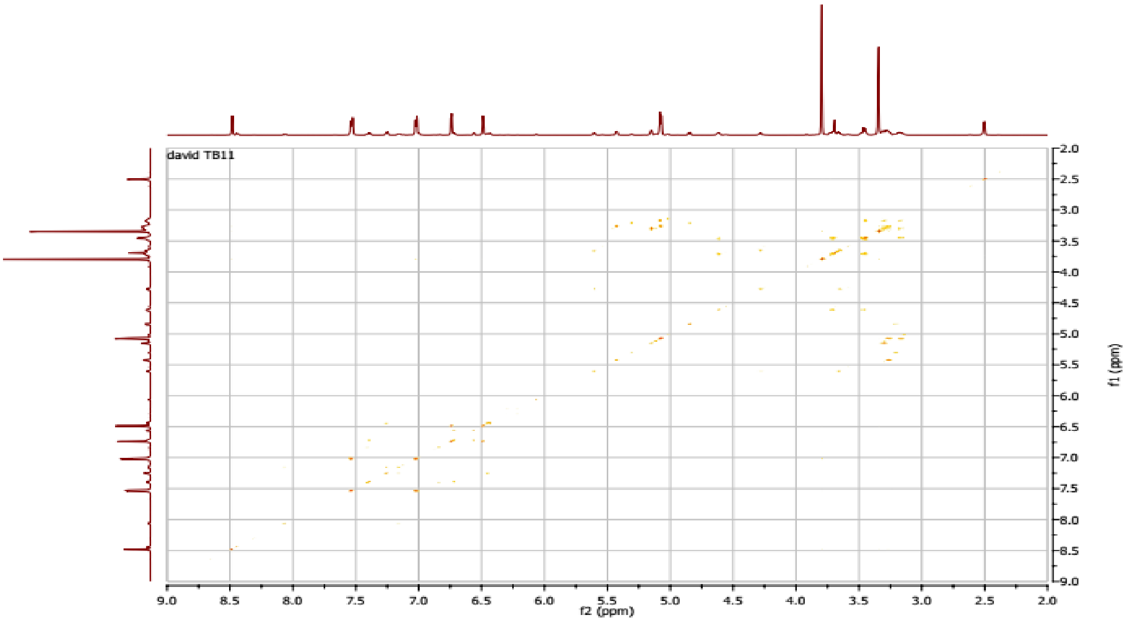

FIGURE S24 : ^{1}H-^{1}H COSY Spectrum of Compound **6**

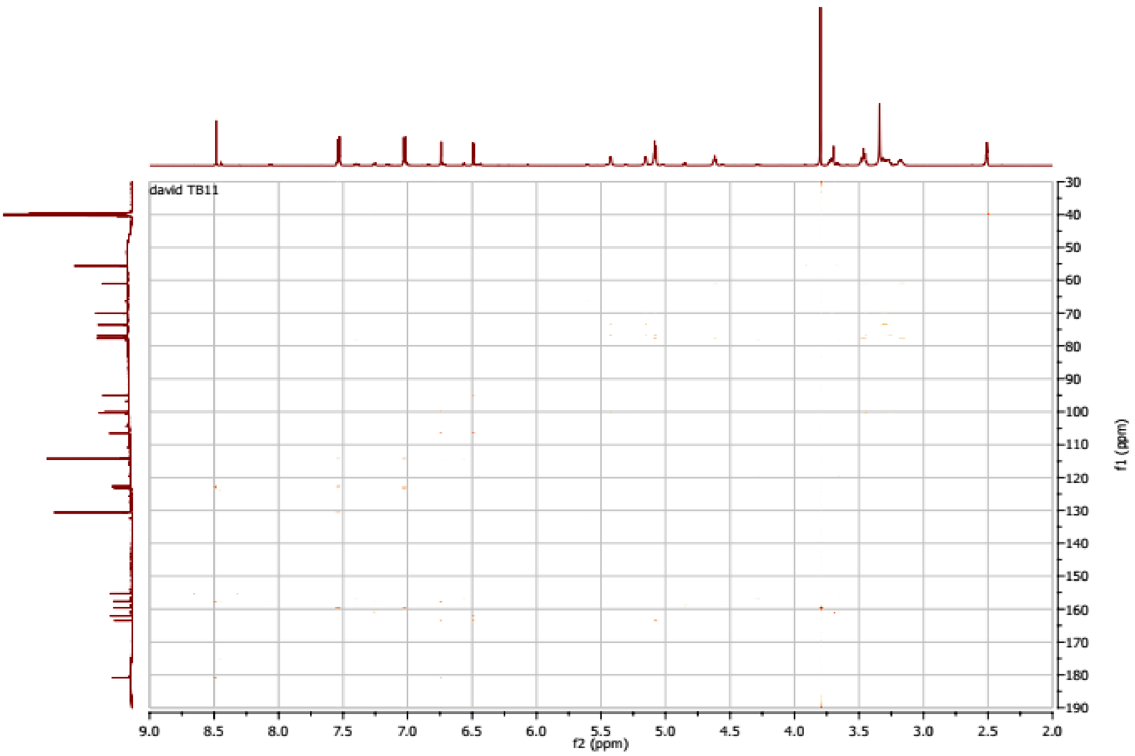

FIGURE S25 : HMBC Spectrum of Compound **6**

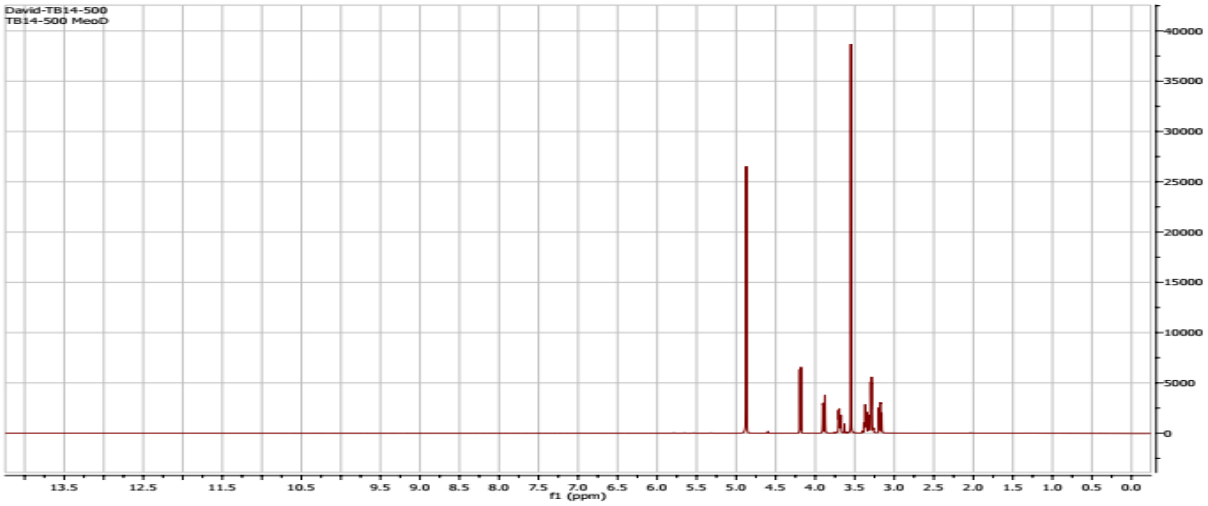

FIGURE S26 : ^{1}H-NMR Spectrum of Compound **7**

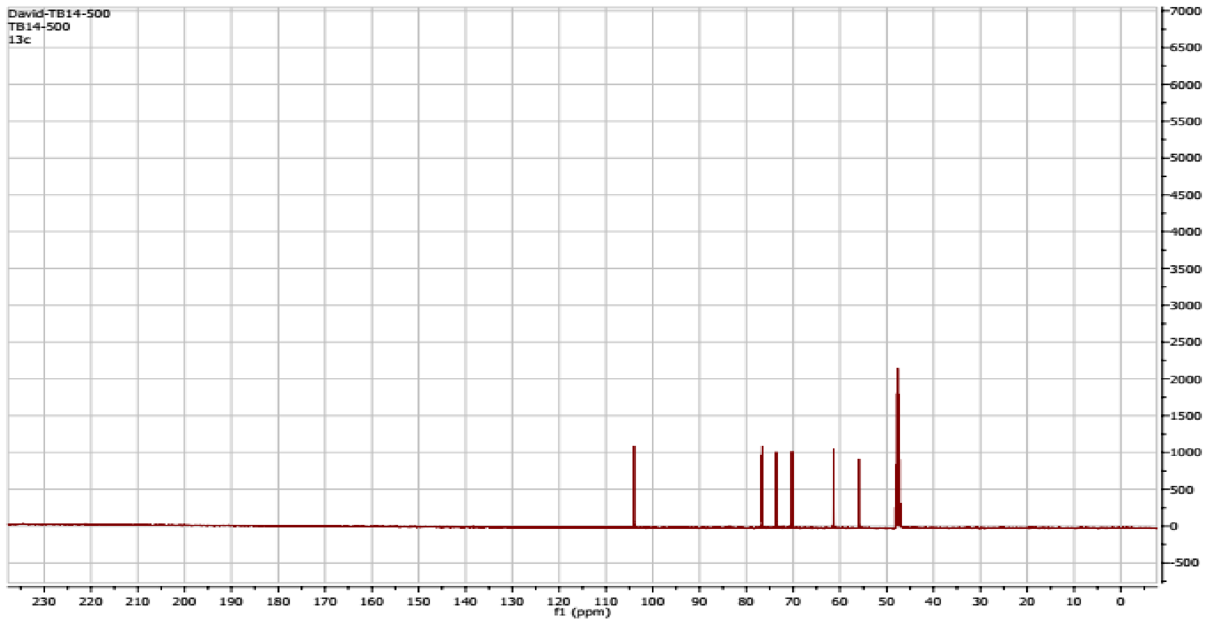

FIGURE S27 : ^{13}C-NMR Spectrum of Compound **7**

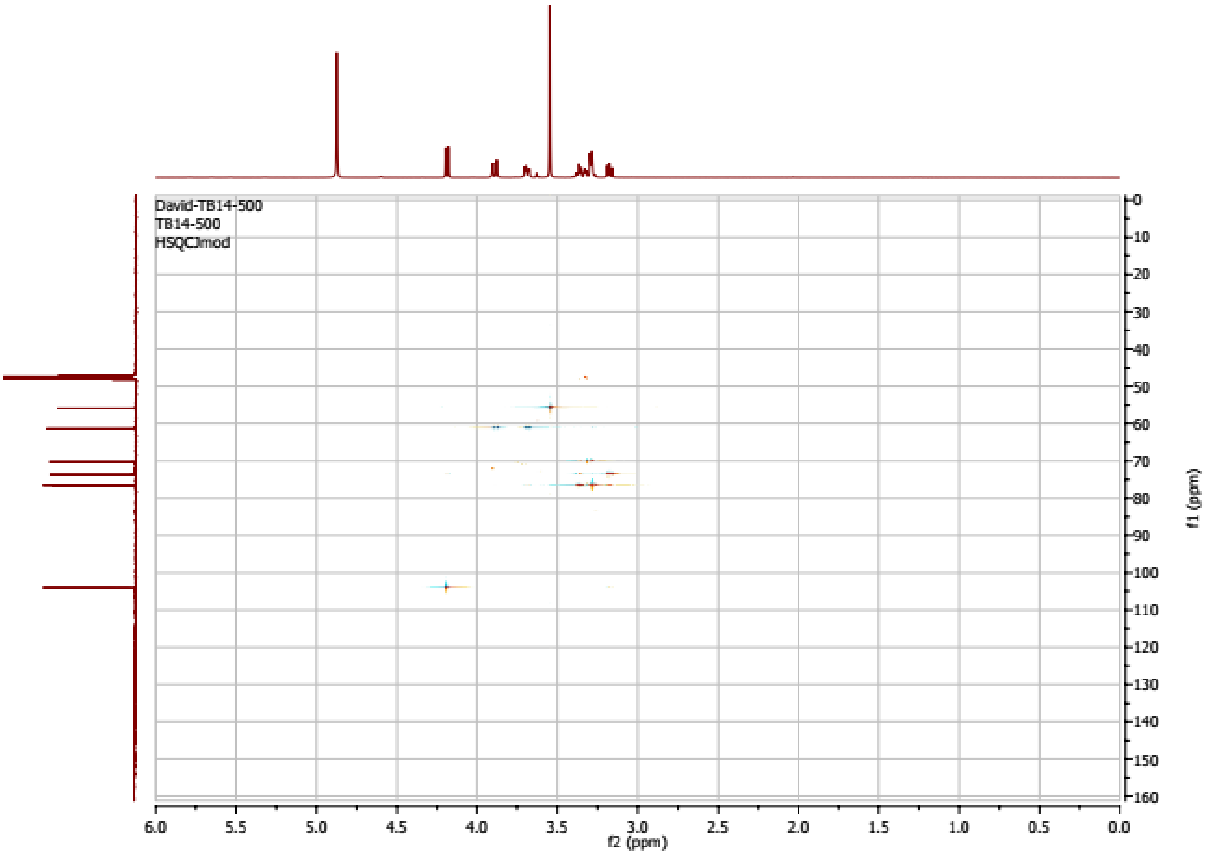

FIGURE S28 : HSQC Spectrum of Compound **7**

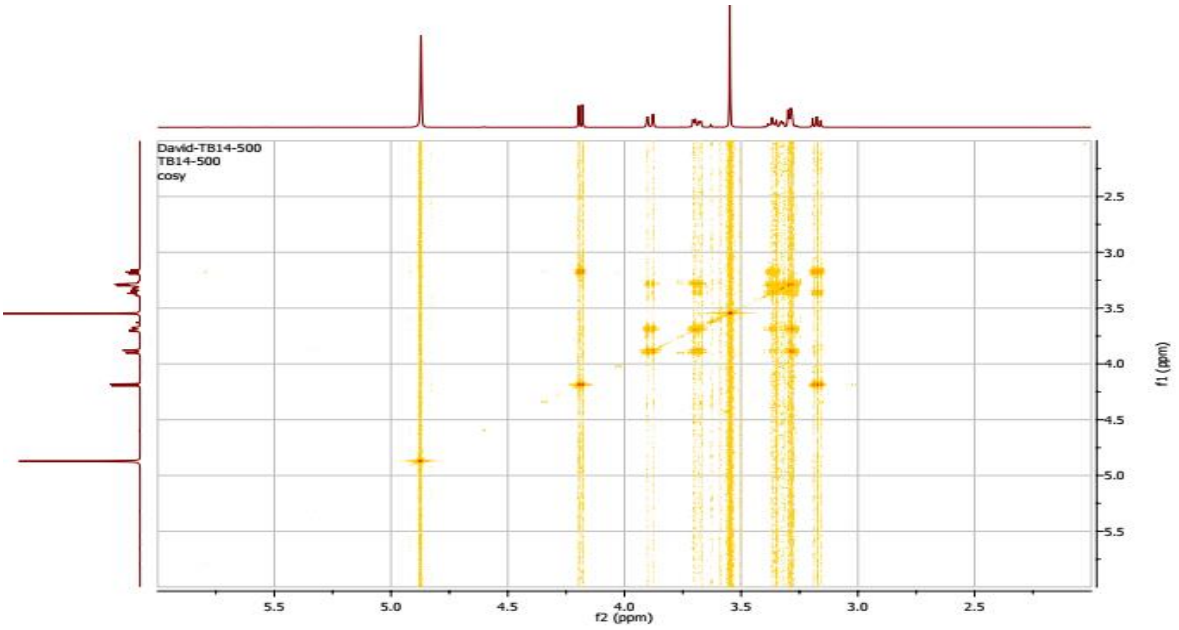

FIGURE S29 : ^{1}H-^{1}H COSY Spectrum of Compound **7**

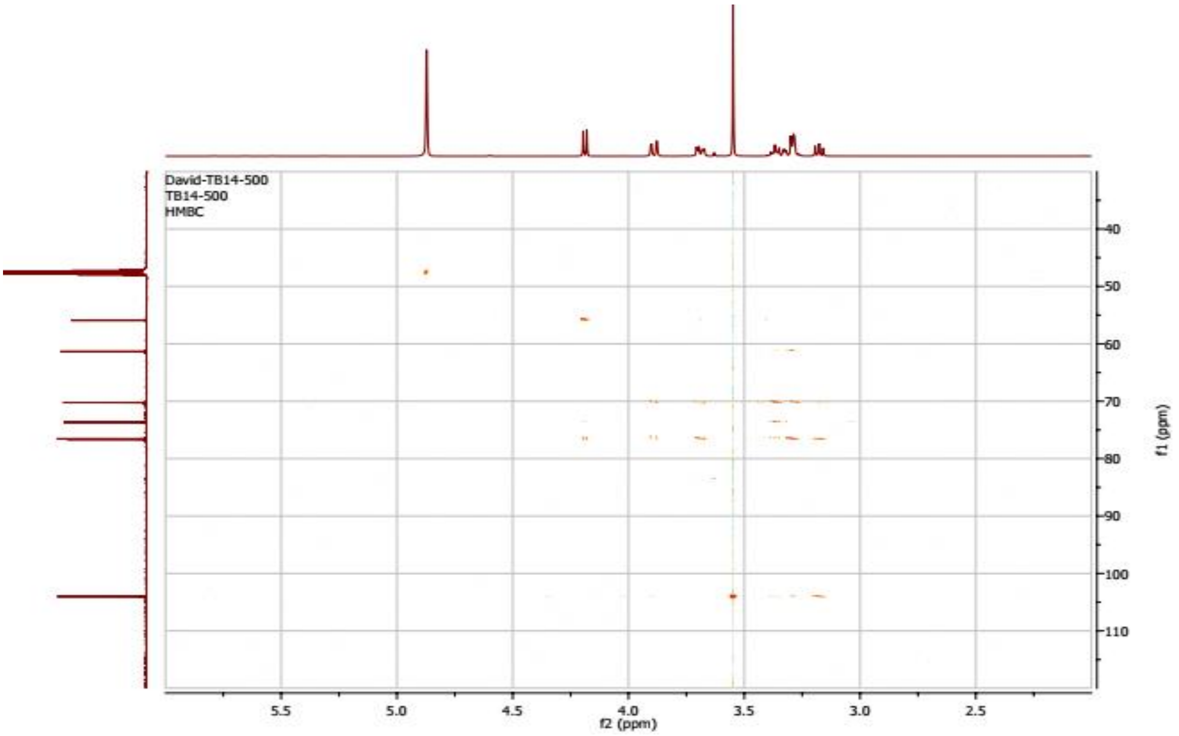

FIGURE S30 : HMBC Spectrum of Compound **7**

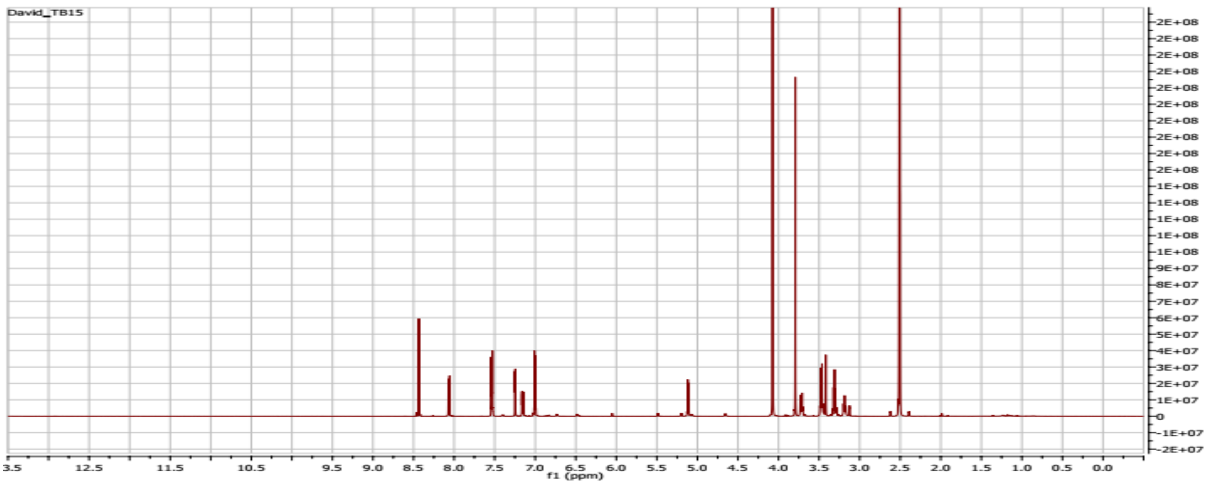

FIGURE S31 : ^{1}H-NMR Spectrum of Compound **8**

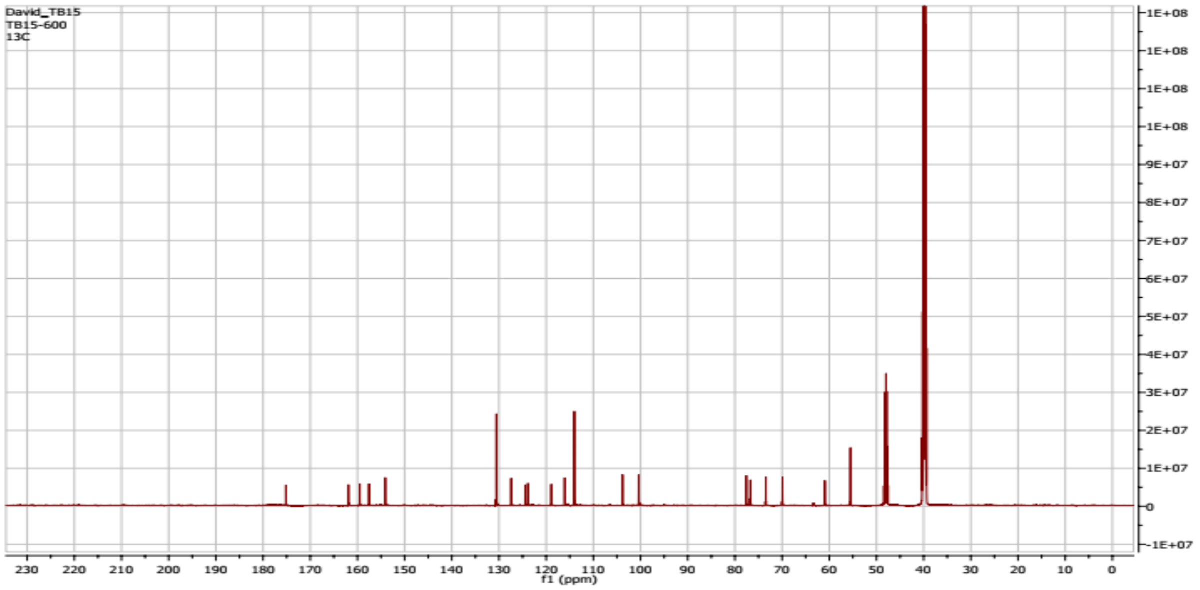

FIGURE S32 : ^{13}C-NMR Spectrum of Compound **8**

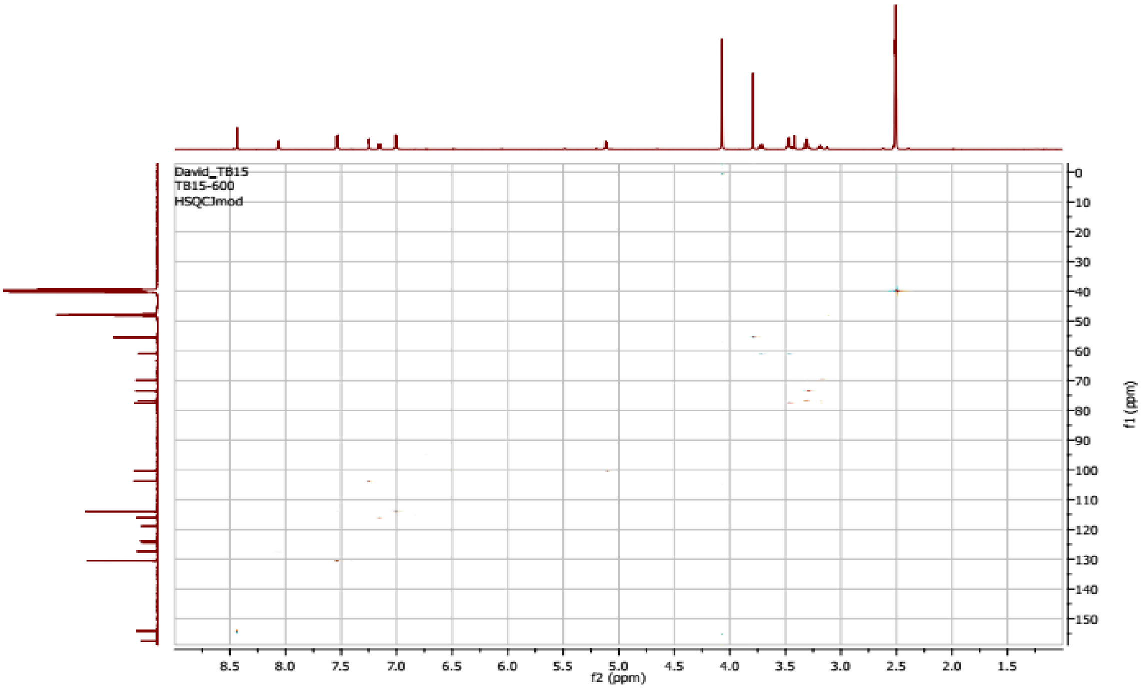

FIGURE S33 : HSQC Spectrum of Compound **8**

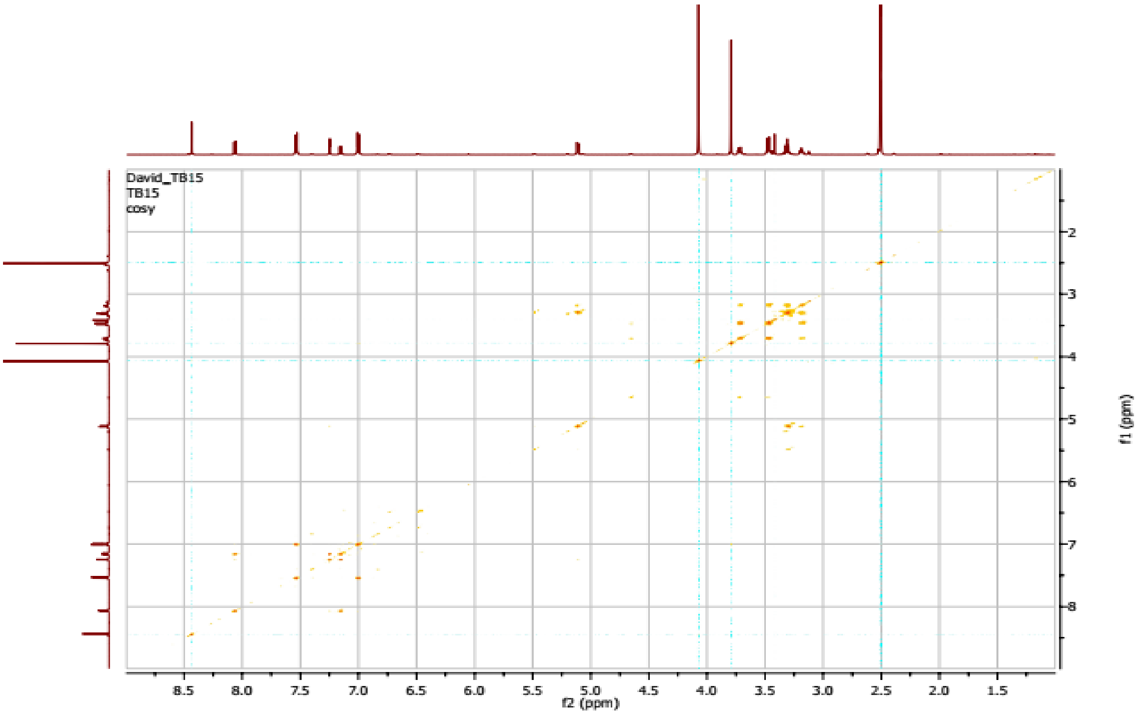

FIGURE S34 : ^{1}H-^{1}H COSY Spectrum of Compound **8**

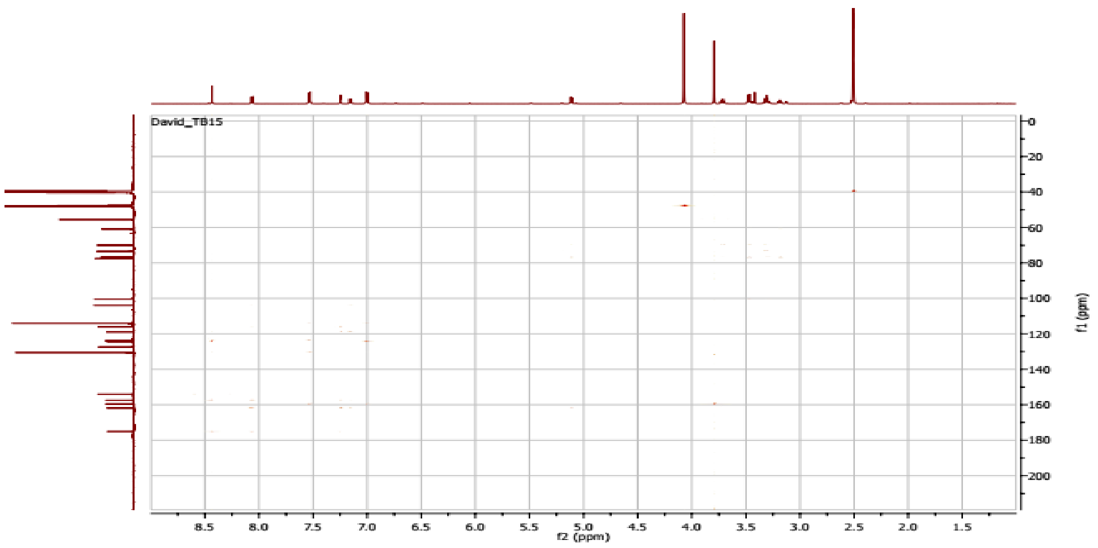

FIGURE S35 : HMBC Spectrum of Compound **8**

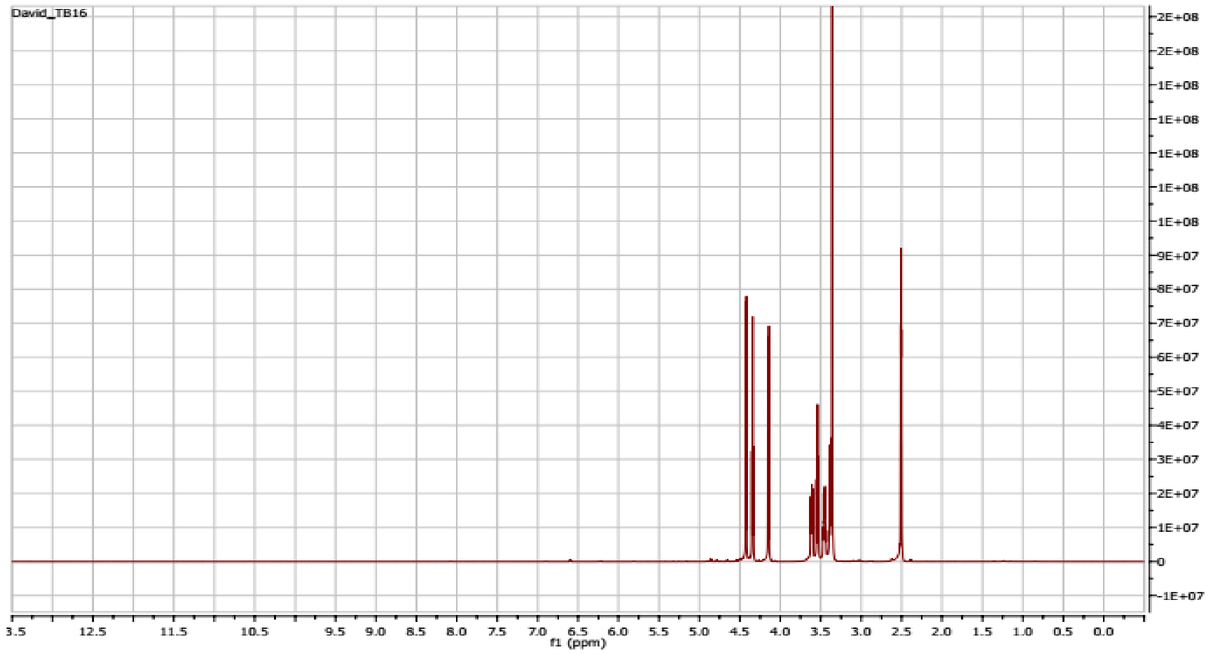

FIGURE S36 : ^{1}H-NMR Spectrum of Compound **9**

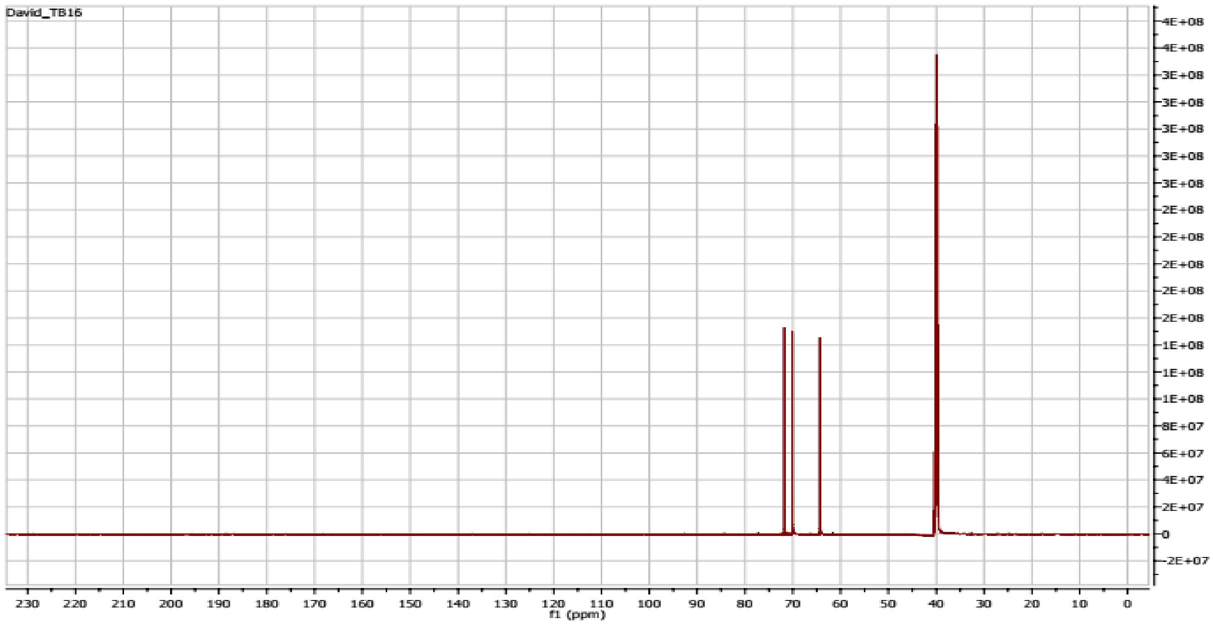

FIGURE S37 : ^{13}C-NMR Spectrum of Compound **9**

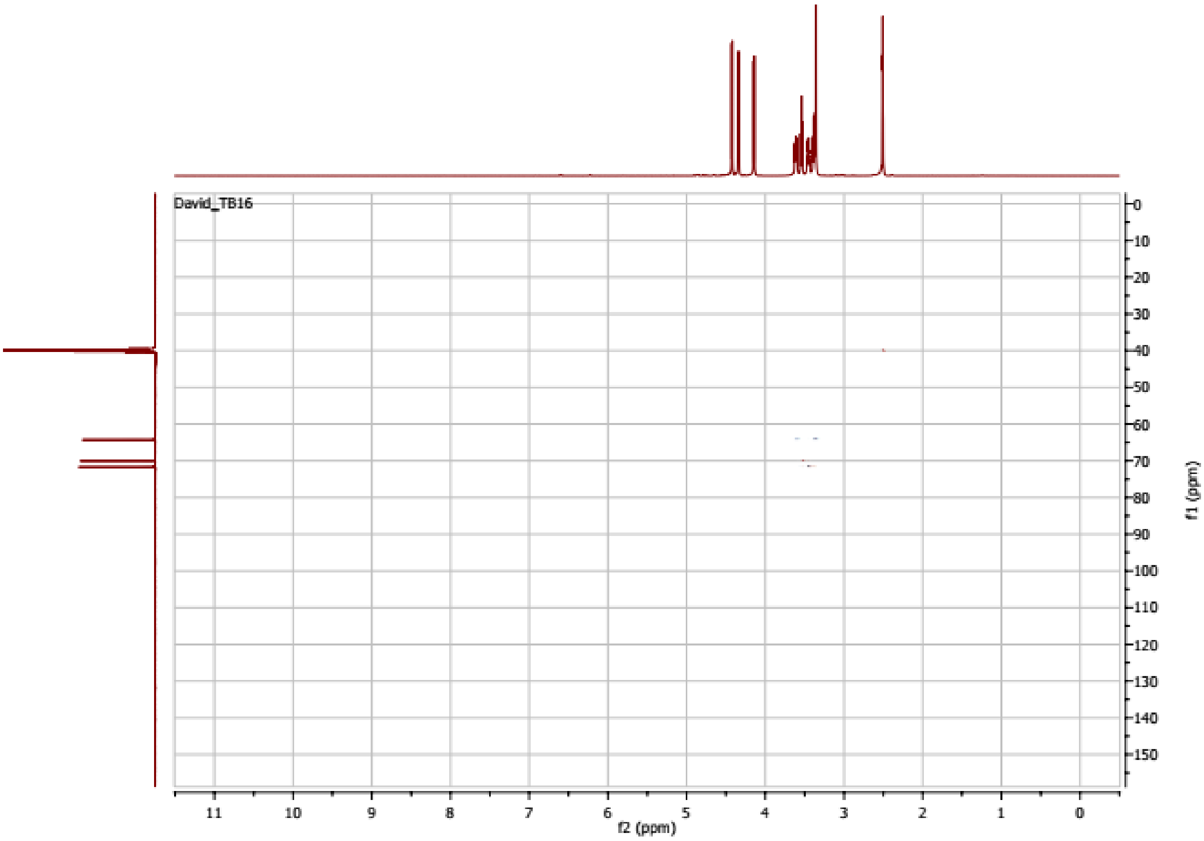

FIGURE S38 : HSQC Spectrum of Compound **9**

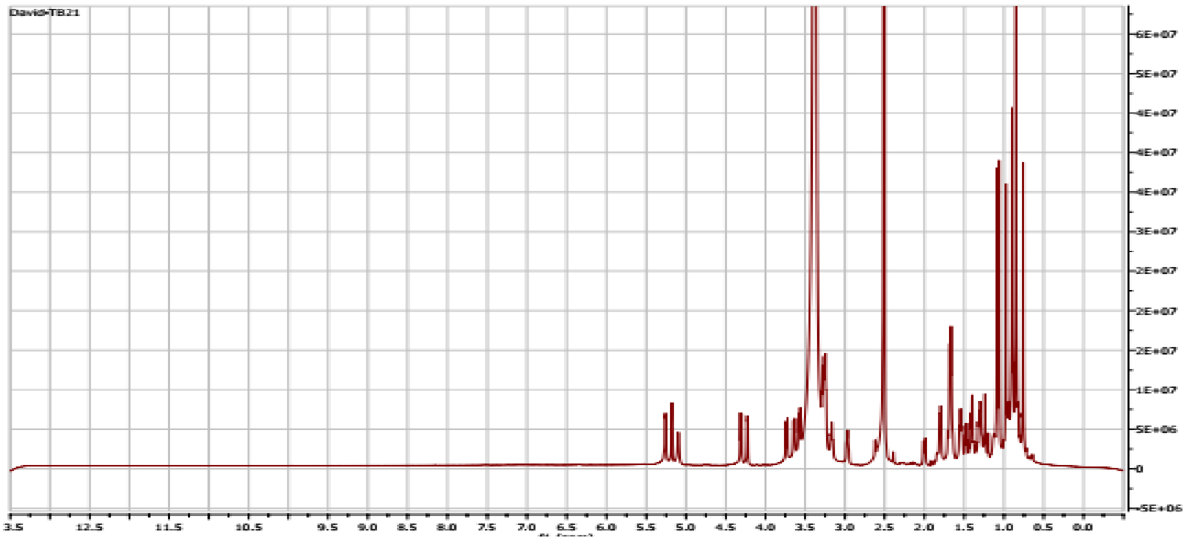

FIGURE S39 : ^{1}H-NMR Spectrum of Compound **10**

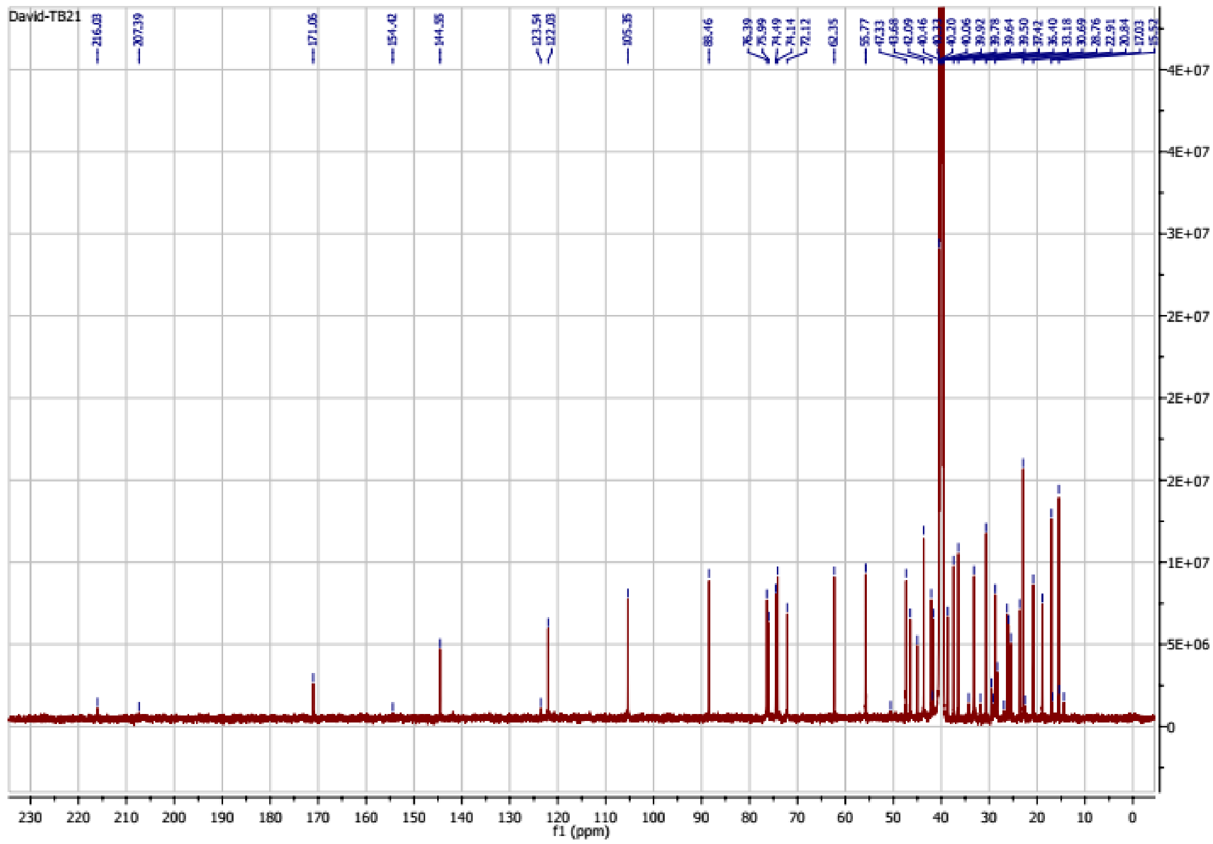

FIGURE S40 : ^{13}C-NMR Spectrum of Compound **10**

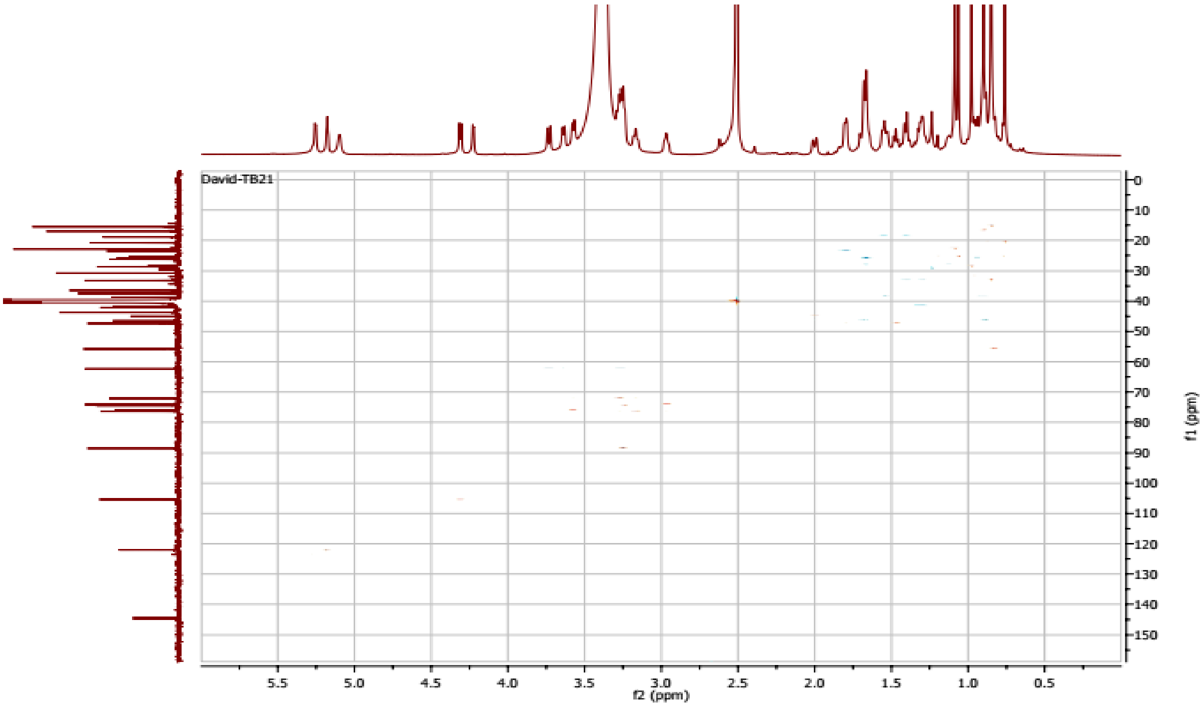

FIGURE S41 : HSQC Spectrum of Compound **10**

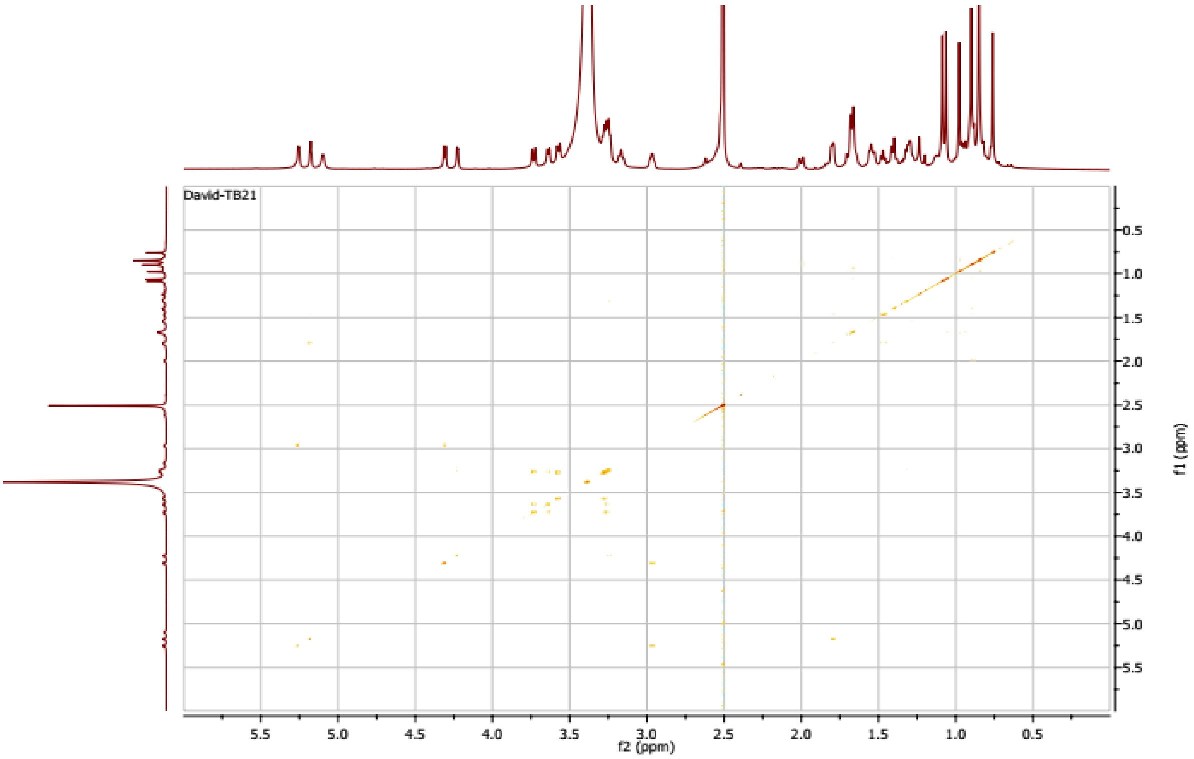

FIGURE S42 : ^{1}H-^{1}H COSY Spectrum of Compound **10**

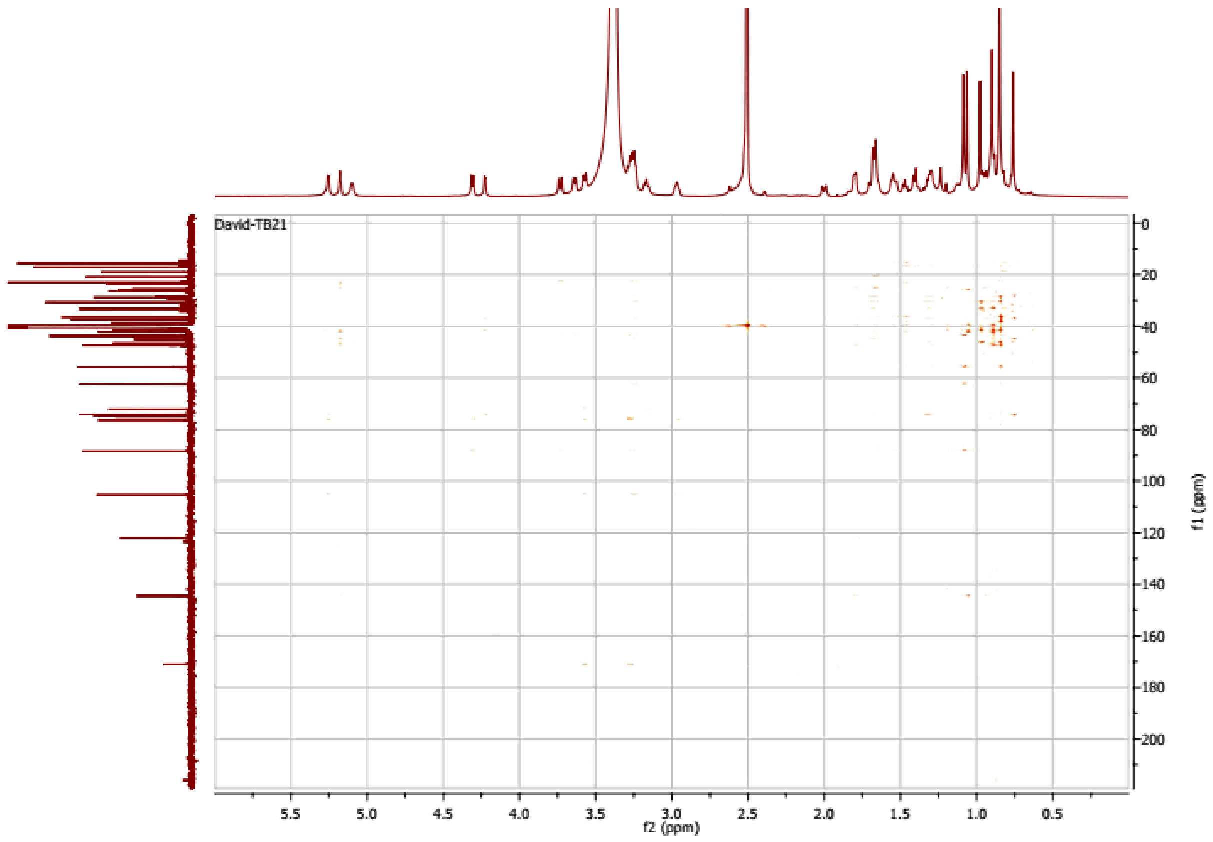

FIGURE S43 : HMBC Spectrum of Compound **10**
